# Improved transition metal photosensitizers to drive advances in photocatalysis

**DOI:** 10.1039/d3sc04580c

**Published:** 2023-11-24

**Authors:** Dooyoung Kim, Vinh Q. Dang, Thomas S. Teets

**Affiliations:** a University of Houston, Department of Chemistry 3585 Cullen Blvd. Room 112 Houston TX 77204-5003 USA tteets@uh.edu

## Abstract

To function effectively in a photocatalytic application, a photosensitizer's light absorption, excited-state lifetime, and redox potentials, both in the ground state and excited state, are critically important. The absorption profile is particularly relevant to applications involving solar harvesting, whereas the redox potentials and excited-state lifetimes determine the thermodynamics, kinetics, and quantum yields of photoinduced redox processes. This perspective article focuses on synthetic inorganic and organometallic approaches to optimize these three characteristics of transition-metal based photosensitizers. We include our own work in these areas, which has focused extensively on exceptionally strong cyclometalated iridium photoreductants that enable challenging reductive photoredox transformations on organic substrates, and more recent work which has led to improved solar harvesting in charge-transfer copper(i) chromophores, an emerging class of earth-abundant compounds particularly relevant to solar-energy applications. We also extensively highlight many other complementary strategies for optimizing these parameters and highlight representative examples from the recent literature. It remains a significant challenge to simultaneously optimize all three of these parameters at once, since improvements in one often come at the detriment of the others. These inherent trade-offs and approaches to obviate or circumvent them are discussed throughout.

## Abstract 2

This perspective focuses on strategies to manipulate and optimize three key determinants of metal-based molecular photosensitizers – the absorption profile, the excited-state redox potentials, and the excited-state lifetime.

## Graphical abstract

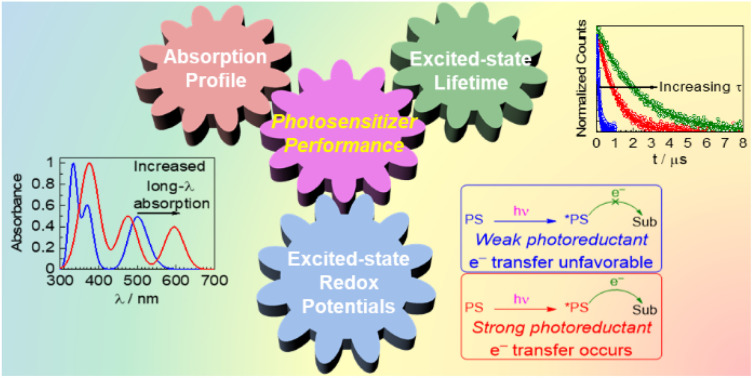

## Introduction and scope

Photosensitizers are molecules that absorb light and initiate either an energy-transfer or redox process, the latter being the focus of this article. We note that it is common in some fields to only use the term “photosensitizer” when energy-transfer processes occur,^1^ but the IUPAC definition is more general^2^ and using the term “photosensitizer” for photoredox reagents is commonplace. There are many classes of compounds that function as photosensitizers,^3^ which span the traditional subfields of organic, inorganic, and organometallic chemistry. While early work was dominated by metal polypyridyl compounds,^4^ many types of organometallic compounds have gained prominence,^5^ with earth-abundant metal complexes^6,7^ and metal-free organic photosensitizers^8–10^ becoming increasingly important in photocatalysis applications as a greater focus on sustainability emerges.

**Fig. 1:**
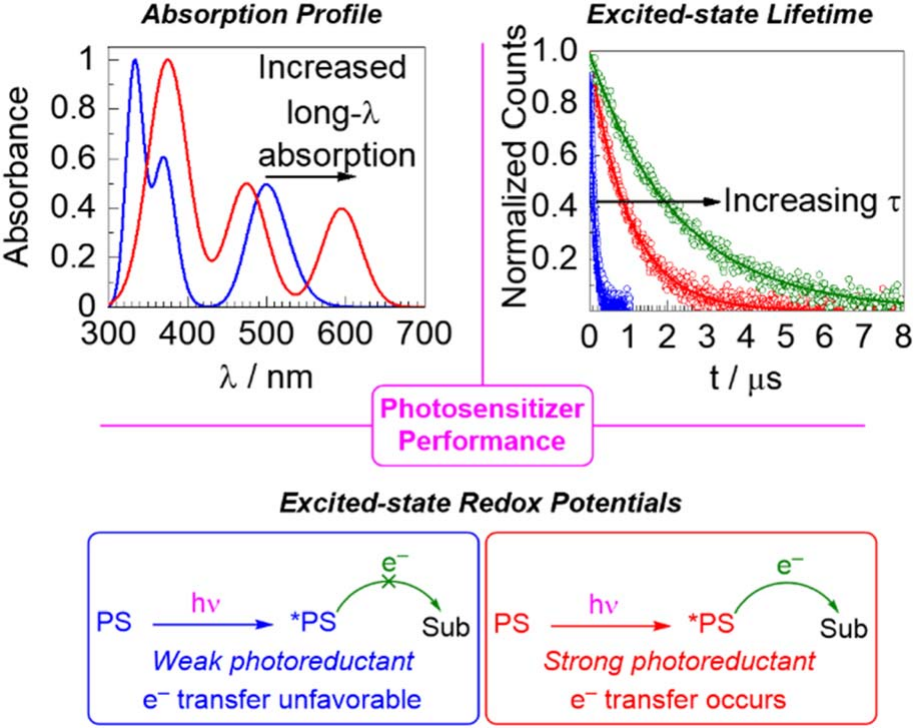
Summary of the three aspects of photosensitizer performance that serve as the basis of this perspective article.

When a photosensitizer is used in a catalytic photoredox reaction, after absorption of light there are several mechanistic pathways possible, and fully understanding the mechanisms of such reactions, which typically involve short-lived radical intermediates, is a complex endeavor.^11–13^ Nevertheless, there are three key parameters that are important to determining the performance of a photosensitizer in a photoredox reaction and the choice of the photosensitizer for a particular application: light absorption, excited-state redox potentials, and excited-state lifetime. These are summarized in Fig. 1, and they represent the thematic organization of this perspective article.

The first characteristic is the light absorption of the photosensitizer, which can be very simply evaluated by recording the photosensitizer's UV-vis absorption spectrum. A major thrust in photocatalysis research is to excite the photocatalyst deeper in the visible or near-infrared region, *i.e.*, to longer wavelengths, which can be accomplished by a combination of new photosensitizer candidates with altered absorption profiles or reaction engineering. The absorption can be specified at a given wavelength by determining the molar absorption coefficient (*ε*) *via* Beer's Law. In the context of solar harvesting for photovoltaics or solar fuel applications, a global quantification of light absorption in terms of its overlap with the solar spectrum is critical and factors directly into the quantum efficiency/yield.^14^ In the field of organic photoredox catalysis a global or quantitative assessment of light absorption is not usually considered, but it is still important to have insight into the photosensitizer's absorption profile to ensure that an appropriate excitation source is used. Moreover, it is ideal that the photosensitizer is excitable in a distinct region of the spectrum from where the substrate(s) or co-catalysts absorb, both to ensure that all photons are collected by the photosensitizer and to prevent deleterious side reactions that may occur when a substrate is directly excited. Control of photosensitizer light absorption also enables photocatalysis *via* red or near-infrared excitation, an emerging trend which has the conceptual appeal of mimicking natural photosynthesis when two photons are used^15,16^ and offers two practical advantages. First, in many cases the photosensitizer's molar absorption coefficient in lower in the red region, particularly when a spin-forbidden transition is used, which is an important consideration in large-scale reactor design that allows a greater penetration depth of light. In addition, there is reduced energy loss when the low-energy excited state can be accessed directly, rather than being generated *via* higher-energy excitation followed by internal conversion and/or intersystem crossing pathways.^17^

Although there are many photochemical transformations that involve excited-state energy transfer elementary steps, in which case the excited-state energies and spin multiplicities of the photosensitizer and substrate are the most important considerations,^18^ a large portion of photochemistry involves excited-state electron transfer steps. In such cases, the photosensitizer either functions as a photoreductant, transferring an electron following excitation, or a photooxidant, accepting an electron while in its excited state. In either case, the excited-state redox potentials of the photosensitizer matter considerably. There must be sufficient thermodynamic driving force for the photoinduced electron transfer step to occur at all, and even if the electron transfer is favorable increasing the driving force can improve kinetics, as per the Marcus relationship.^19^ As such, perturbing excited-state redox potentials to more extreme values can promote transformations on redox-inert substrates such as ketones, imines, and unactivated aryl halides.

Finally, the third major theme of this perspective centers on the importance of the photosensitizer's excited-state lifetime in photocatalytic applications. Since photocatalyzed transformations inevitably involve a bimolecular reaction in the excited state, the lifetime of this state must be long enough to permit diffusion of the reaction partner and the electron-transfer (or energy-transfer) step to occur. While at a practical level increasing the excited-state lifetime past a certain point does not continually improve performance, photosensitizers with short excited-state lifetimes do require higher reaction concentrations and, because of the lower quantum yields for their excited-state bimolecular transformations, often involve longer reaction times and lower photon efficiency. Excited-state lifetime is a particular challenge in several emerging classes of earth-abundant metal photosensitizers and photosensitizers with low-energy excited states capable of efficient solar harvesting.

Additionally, it is worth noting that other factors such as chemical stability, photostability, quantum yield, and cage escape should also be considered, although those factors won't be discussed in detail in this paper. Chemical stability and photostability are important for ensuring the longevity of a photosensitizer in a catalytic reaction and allowing low catalyst loadings to be used. Relatedly, as many applications of photosensitizers involve redox chemistry, the different redox states that are involved must also be reasonably stable, to ensure that they live long enough for diffusional reactivity and ensure that the catalytic cycle can be efficiently closed. The quantum yields of energy-transfer or electron-transfer elementary steps are related to the rate of a photocatalytic reaction and dictate the photon flux that is needed to maintain a suitable turnover frequency. Another important consideration, which directly relates to quantum yield, is that after the electron transfer between the photosensitizer and the substrate occurs, the radical ions initially form an ion pair.^20,21^ Dissociation of this ion pair, *i.e.* cage escape, needs to occur before the oxidized or reduced species can diffuse and react further. Thus, even in situations where the quenching event is very efficient, many such events can be nonproductive if cage escape is not also considered.

This perspective article summarizes recent developments in all three of the above-mentioned themes, including our own work in these areas. We have chosen the three major themes (absorption profile, lifetime, and excited-state redox chemistry) because they are readily optimized by synthetic modification and capture the emphases of our own work in this area, but the other factors mentioned in the previous paragraph are also critically important. This article is not designed to be comprehensive and does not include a large catalogue of numeric metrical parameters, but rather focuses on fundamental chemistry strategies for addressing and improving all three of the major characteristics listed in Fig. 1. We also note that scaling relationships result in inherent trade-offs between these three criteria, so we will point out situations where optimization of one of them may be detrimental to one or both of the others. Work from the past five years is most strongly emphasized, although some of the discussed approaches have origins that predate that time period. For more comprehensive review articles that cover classes of photosensitizers and their properties,^3,5,6,9,22^ or applications in photoredox catalysis,^23^ we refer the readers to other recent reviews.

## Improved light absorption

### Ligand modification of heteroleptic bis-chelate Cu(i) chromophores

In this section, we focus on strategies to use longer-wavelength light to excite molecular photosensitizers, which involve both molecular design strategies to access photosensitizers with extended visible absorption and/or new mechanistic and methodological approaches that enable lower-energy photons to be used. There are several motivations to use longer-wavelength light in photocatalytic transformations, which include improved solar harvesting in the context of solar energy conversion, reducing or eliminating wasteful energy-loss processes that often occur following excitation to higher-energy states, and an increased penetration depth of red to near-infrared light. As demonstrated in this section, there are a few distinct approaches that permit longer-wavelength excitation of photosensitizers in the context of photocatalysis.

**Fig. 2:**
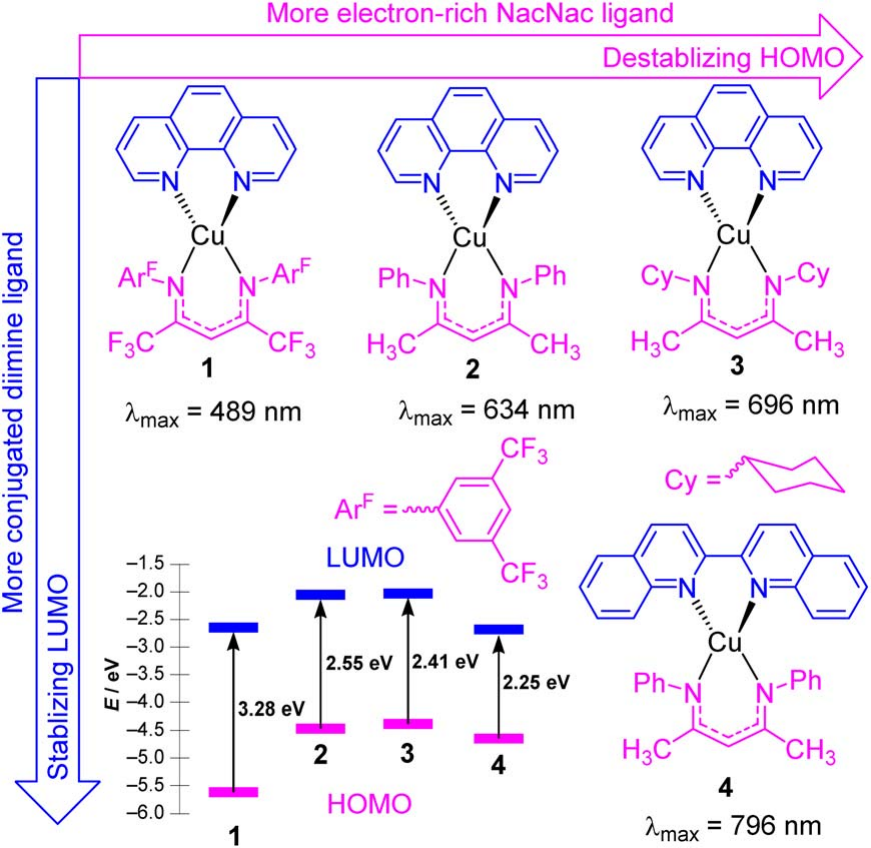
Summary of heteroleptic Cu(N^N)(NacNac^R^) complexes and their frontier orbital energy levels. Frontier orbital energies were determined by DFT.

Copper(i) photosensitizers have become increasingly well-studied, given the low cost and high abundance of copper relative to precious metals, and the advantages offered by the d^10^ configuration of copper(i). Homoleptic copper bis-diimine complexes are effective in many applications,^24–26^ and cationic heteroleptic complexes with mixed diimine/diphosphine coordination can offer the advantage of substantially longer lifetimes.^27–29^ However, neither of these well-known classes of copper(i) charge-transfer chromophores have particularly intense long-wavelength absorption, the former mainly absorbing in the blue and green regions, and the latter in the UV and deep blue. There have been some recent advances in the design of copper(i) complexes with longer-wavelength absorption, including a series of carbene-supported compounds with tunable charge-transfer absorption throughout the visible region.^30^ In some of our group's most recent work, we have introduced four heteroleptic bis-chelate copper(i) chromophores (1–4) as illustrated in Fig. 2. These chromophores consist of the neutral diimine ligands 1,10-phenanthroline (phen) and 2,2′-biquinoline (biq), combined with anionic β-diketiminate (NacNac^R^) ligands.^31^ DFT calculations reveal that the HOMO is delocalized between Cu and the NacNac^R^ ligand, in line with our previous studies on iridium(iii) complexes containing NacNac^R^ ligands.^32,33^ Conversely, the lowest unoccupied molecular orbital (LUMO) is predominantly localized on the diimine ligand. This arrangement provides two potential sites for controlling the HOMO–LUMO gap and the corresponding charge-transfer absorption band through ligand modification. The electronic effects of the β-diketiminate ligands on the energy levels of the frontier orbitals were investigated in complexes 1–3. The results demonstrate that more electron-rich NacNac^R^ ligands (NacNac^Cy^ > NacNac^Me^ > NacNac^F18^) lead to the destabilization of the HOMO. As shown in Fig. 2, the HOMO energies significantly increase from −5.62 eV (complex 1) to −4.32 eV (complex 3), whereas the LUMO energies show minimal dependence on the substituents of the NacNac ligand. On the other hand, altering the diimine ligand (complexes 2 and 4) impacts the LUMO energies of these copper(i) complexes. The LUMO energy decreases by 0.45 eV with only a slight change in the HOMO energy (Fig. 2) when phen (complex 2) is replaced with biq (complex 4). As a result, all four complexes (1–4) display low-energy charge transfer bands in the visible to near-infrared (NIR) regions. The combination of the electron-rich NacNac^Me^ ligand and π-extended biq ligand (complex 4) exhibits the longest peak wavelength for the charge-transfer band (796 nm), surpassing the wavelengths observed in complexes 3 (696 nm), 2 (634 nm), and 1 (489 nm). From time-dependent density-functional theory (TD-DFT), these low-energy transitions can be assigned as a mixture of metal-to-ligand charge transfer (MLCT) and ligand-to-ligand charge transfer (LLCT). Unfortunately, complexes 1–4 do not exhibit emission in solution, and our attempts in photocatalysis have been unsuccessful, suggesting a short excited-state lifetime for these compounds. Ongoing work addresses these limitations of this promising class of chromophores, to allow us to leverage their intense, long-wavelength visible absorption in photocatalysis applications.

### HOMO inversion

**Fig. 3:**
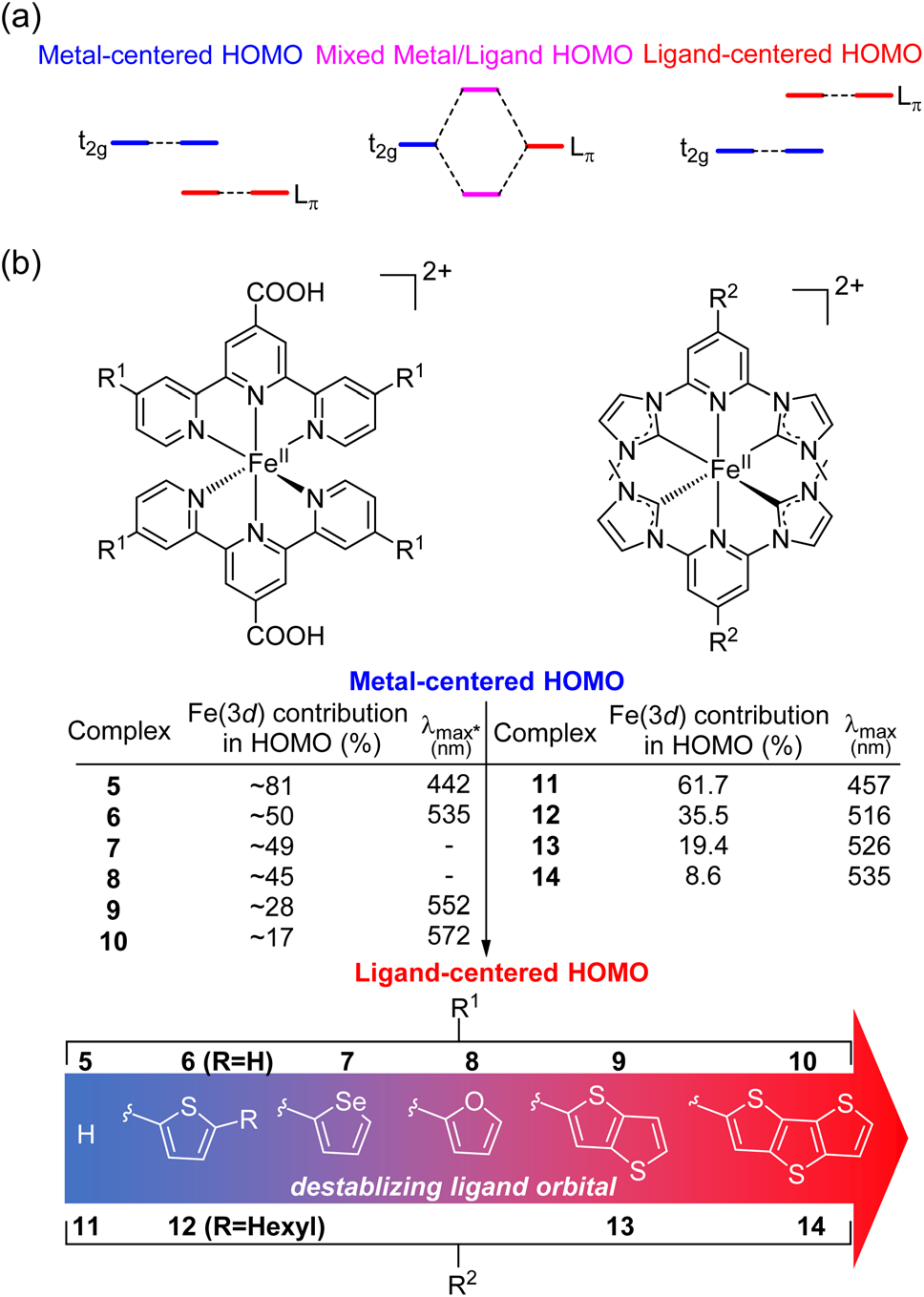
(a) Simplified HOMO energy levels depicting the “HOMO inversion” strategy. (b) Summary of representative bis(tridentate) iron(ii) complexes, demonstrating the correlation between the substitution of the strong donor group with enhanced conjugation and the resultant absorption wavelength. *The *λ*_max_ values in the table are the calculated values for complexes 5–10.

Recently, there has been growing interest in improving the light absorption of iron(ii) polypyridine complexes *via* the “HOMO inversion” strategy. Traditionally, the HOMO of iron(ii) polypyridine complexes mainly consists of metal t_2g_ orbitals, with a large energy gap separating them from the lower-energy ligand π orbitals (Fig. 3a). By destabilizing the ligand π orbitals, the interaction between the t_2g_ orbital and ligand π orbitals can be increased, leading to a dramatic destabilization of the HOMO. Further destabilization of the ligand π orbital would cause a complete inversion of the HOMO, making it ligand-centered instead of metal-centered (HOMO inversion). The concept of HOMO inversion has been outlined in a few different studies over the past decade or so,^34–36^ and a computational study was recently done by Mukherjee and co-workers (Fig. 3b).^37^ This study focused on the effect of the substitution on the 2,2′;6′,2′′-terpyridine (tpy) ligand of [Fe(tpy)_2_]^2+^ complexes. It was observed that the substitution of a donor group with more π conjugation on the tpy ligand would significantly destabilize the ligand orbitals. As a result, strong mixing between filled metal t_2g_ orbitals and filled ligand orbitals was noted through the decrease of electron density on Fe (from 81% for 5 to approximately 50% for 6–8). Due to the strong mixing between the t_2g_ orbitals and the ligand π orbitals, the HOMO was significantly destabilized, and the calculated absorption spectra were dramatically red shifted in complex 6. Further destabilization of π-donor ligand orbitals through the extension of π-conjugation of the donor group reduced the electron density on the Fe and the HOMO became ligand-centered (complexes 9 and 10) with further improvement in light absorption. Very recently, Marri and co-workers provided experimental evidence supporting the concept of HOMO inversion in their study on pyridyl *N*-heterocyclic (NHC) iron(ii) complexes (11–14), specifically investigating the impact of extending the π conjugation of thiophenes (Fig. 3b).^38^ In the same vein as the terpyridine analogues (5–10), extending the π-conjugation of the thiophene substituents resulted in a red shift of the absorption spectrum. Another recent representative example of HOMO inversion is found in iron complexes with benzannulated amido pincer-like ligands developed by Braun and co-workers.^39^ These complexes exhibit strong absorption in the visible light region tailing out to 850 nm. Additionally, the same group also developed an alternative strategy to HOMO inversion by keeping the ligand orbital unchanged and stabilizing the metal d orbitals by changing the metal (Co, Ni, Zn, and Ga).^40^ Although the electronic structure of complexes was changed by altering the metal center, with negligible contribution of the metal observed in the HOMO for these complexes, similar panchromatic absorption was observed.

### Spin-forbidden S_0_ → T_1_ excitation

**Fig. 4:**
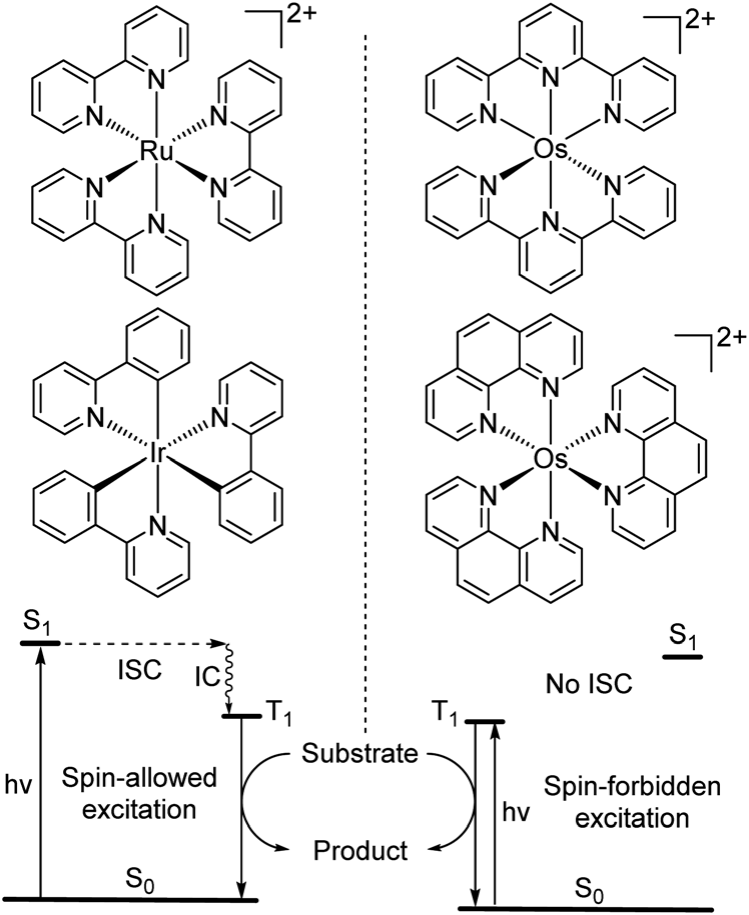
Photocatalysis *via* conventional S_0_ to S_1_ absorption and spin-forbidden S_0_ to T_1_ absorption (ISC = intersystem crossing and IC = internal conversion).

**Fig. 5:**
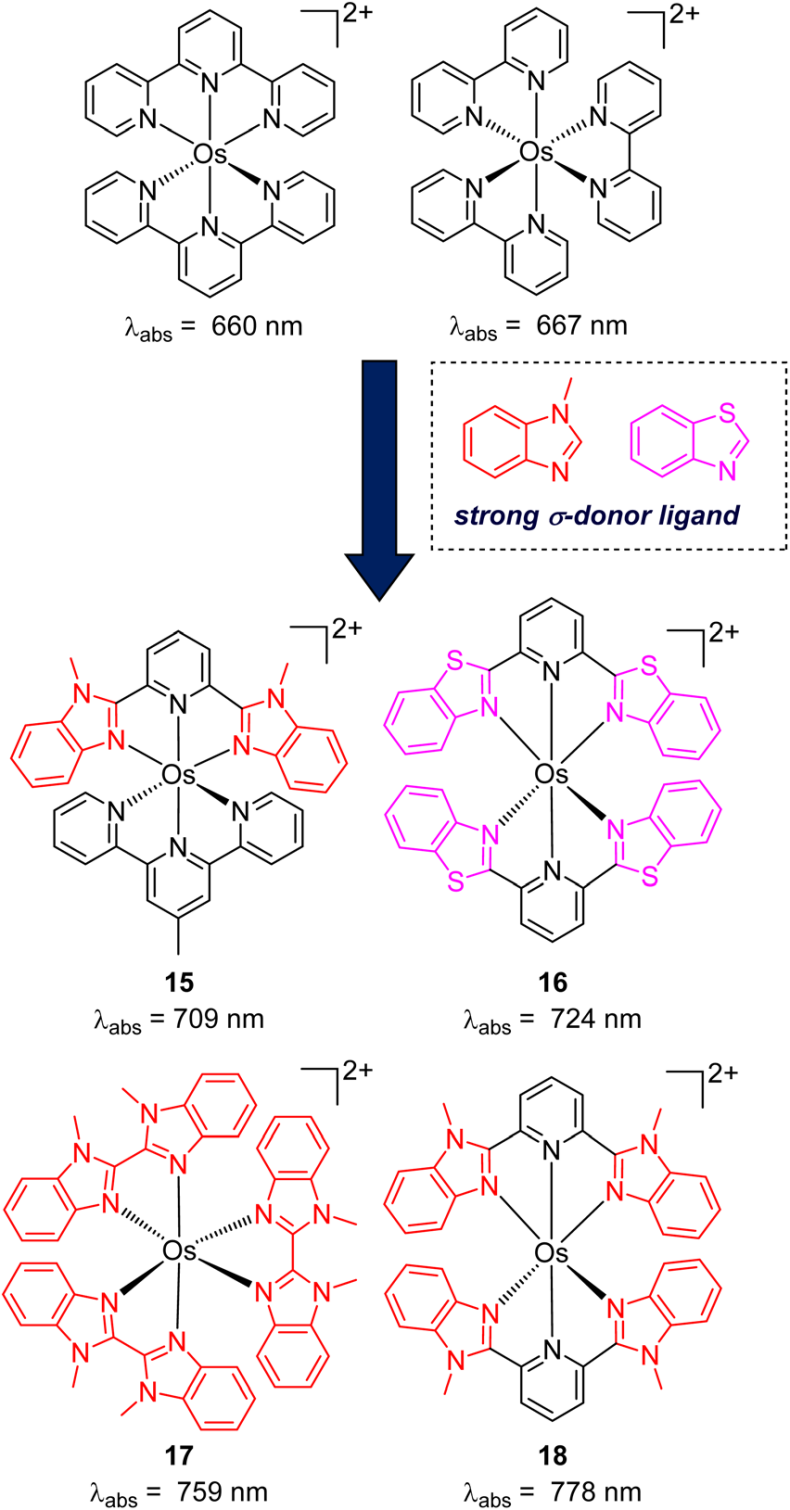
The improvement of light absorption *via* introducing a strong σ-donor ligand into osmium(ii) complexes.

While the modification of ligands to engineer the HOMO–LUMO gap has been an effective approach, these strategies could have some drawbacks, as many of the compounds described above have short excited-state lifetime and/or insufficient reducing or oxidizing power in the excited state, limiting their applicability. An alternative approach to extend the wavelength of light absorption is to target the direct singlet to triplet excitation, which not only allows longer-wavelength excitation but also avoids the energy loss that occurs during intersystem crossing (Fig. 4). Fundamentally, the transition from the singlet ground state to the triplet excited state is rare because it is a spin-forbidden process. However, photosensitizers based on heavy transition metals with a large spin–orbit coupling constant can be directly excited to the triplet state. The advantage of this approach in improved long-wavelength light absorption was clearly shown in recent studies by the Rovis group. In these studies, a series of osmium(ii) complexes with variable polypyridyl analogues were synthesized.^17,41^ Most complexes exhibit direct triplet absorption in the deep red or NIR region (600–800 nm). Through these studies, the photophysical properties of osmium complexes could be enhanced by using strong σ-donor ligands through the destabilization of the HOMO. For instance, ligands based on methylbenzimidazole or methylbenzothiazole units could induce longer triplet absorption wavelengths (complexes 15–18) compared to [Os(tpy)_2_](PF_6_)_2_ or [Os(bpy)_3_](PF_6_)_2_ (Fig. 5, bpy = 2,2′-bipyridine).^17,41,42^ Additionally, to improve the molar absorption coefficient, the use of extended ligand conjugation was considered as a potential approach.^43^ In addition to osmium-based complexes, the Rovis group recently developed a series of Ir(iii) photocatalysts that could undergo spin-forbidden excitation.^44^ This suggests a broad range of applications for this approach in various heavy metal complexes.

The Rovis group also reported the pioneering work on photocatalytic applications of osmium(ii) polypyridyl complexes akin to those in Fig. 5. They used near-infrared (NIR) or deep red irradiation for a wide range of transformations such as photoredox, photopolymerization, and metallaphotoredox.^17,45^ After the work of Rovis's group, several red-light-induced photoreactions based on osmium complexes have been developed. For instance, Ogbu and co-workers utilized [Os(bptpy)_2_](PF_6_)_2_ (bptpy = 4′-(4-bromophenyl)-2,2′:6′,2′′-terpyridine) as a photocatalyst and 1-hydroxy-1,2-benziodoxol-3(1*H*)-one as an oxidant for the decarboxylation of oxamic acid under NIR light irradiation.^46^ Very recently, Cabanero and co-workers described a NIR light-controlled Ru-catalyzed olefin metathesis reaction using [Os(phen)_3_](PF_6_)_2_ as a photocatalyst to activate the Ru co-catalyst.^47^ Regarding biological applications, osmium(ii) complexes were successfully applied for the activation of aryl azides under red light irradiation, enabling targeted labelling applications.^48^ All of these studies demonstrated the superiority of osmium complexes under red or NIR irradiation compared to common photocatalysts, which require higher-energy light sources, due to the better penetration of red to NIR light in various materials and tissues.

### Multiphoton catalysis

**Fig. 6:**
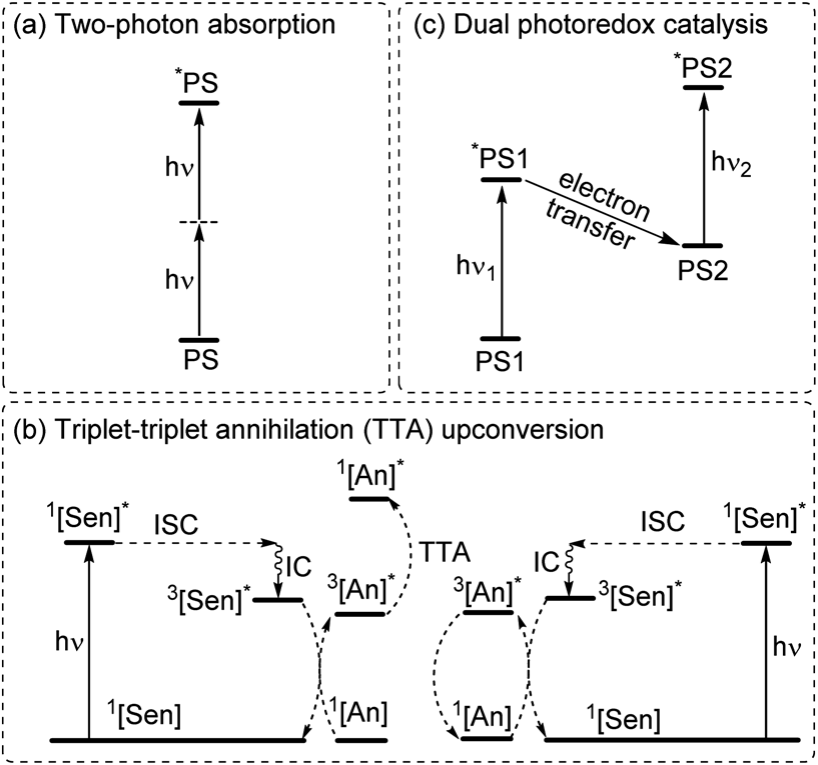
Summary of strategies for achieving longer wavelengths in the activation of photoreactions through multiphoton absorption. (a) Two-photon absorption. (b) Dual photoredox catalysis. (c) Triplet–triplet annihilation upconversion. (PS = photosensitizer, Sen = sensitizer, An = annihilator, ISC = intersystem crossing, and IC = internal conversion, and PS1 and PS2 can be the same or different photosensitizers).

To increase the wavelength of light used to trigger photoreactions, the concept of multiphoton catalysis has emerged, wherein multiple low-energy photons are combined to access a higher-energy level. Three main approaches that have been used in multiphoton catalysis are displayed in Fig. 6, including two-photon absorption (TPA), triplet–triplet annihilation (TTA) upconversion, and dual photoredox catalysis. We also note that multiphoton approaches can be used to generate exceptionally strong excited-state redox reagents, as described in the later section on excited-state redox potentials.

**Fig. 7:**
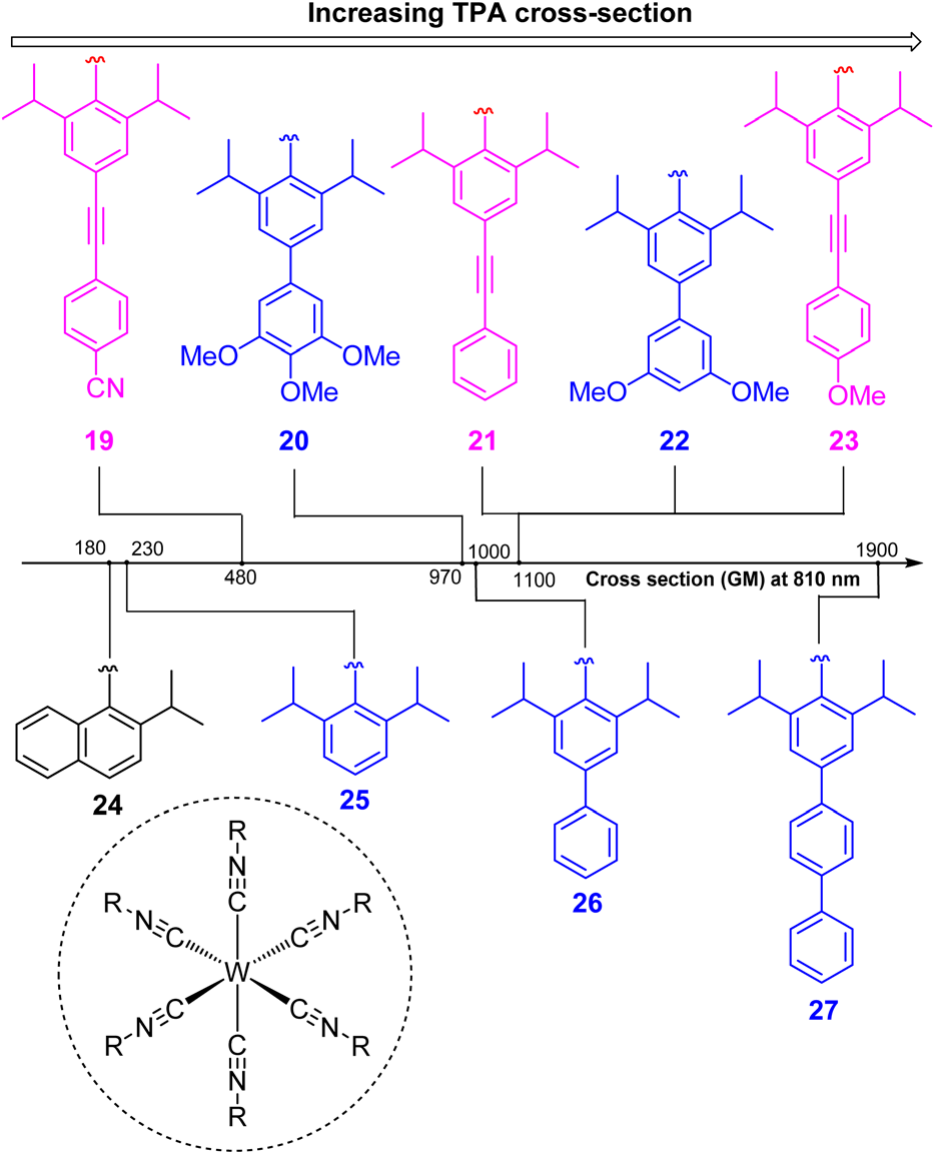
The improvement of the TTA cross section of tungsten complexes *via* modifications to aryl isocyanide ligands.

In two-photon absorption, a photosensitizer simultaneously absorbs two photons to access a higher excited state (Fig. 6a). Through this mechanism, the photocatalyst can use photons with half of the energy required for one-photon absorption. The main limitation of TPA is the requirement of high-intensity light sources due to their weak absorption. To quantify TPA, researchers commonly refer to the TPA cross section, usually reported in Göppert-Mayer (GM) units (1 GM = 10^−50^ cm^4^ s per photons per molecule).^49^ To enhance the TPA cross section, symmetry plays an important role. In short, symmetry-based selection rules for two-photon absorbers dictate that centrosymmetric molecules will have higher TPA cross sections, all else being equal.^49^ In addition to symmetry, two common approaches are widely employed to further improve the TPA cross section: (i) extension of π-conjugation and (ii) substitution of electron-donating groups. An early example of enhancing TPA through the extension of π-conjugation was observed in Ir complexes, where the cross section significantly increases from 17 GM in [Ir(ppy)_2_(acac)] (ppyH = 2-phenylpyridine and acac = acetylacetonate) to 44 GM in Ir(4-pe-2-ppy)_2_(acac) (4-pe-2-ppyH = 4-phenylethynyl-2-phenylpyridine).^50^ Recently, a series of tungsten(0) aryl isocyanides were described by Fajardo and co-workers (Fig. 7).^51^ In this work, the TPA cross section of tungsten complexes was strongly responsive to the structure of the aryl isocyanide ligand. Increasing conjugation in oligoaryl isocyanides in general increases the TPA cross section (24–27, bottom part of Fig. 7), and the values are further modulated by electron-donating and electron-withdrawing groups on the periphery (19–23, top part of Fig. 7). Importantly, these complexes are active in photoredox transformations when excited with femtosecond-pulsed 810 nm irradiation, with TPA populating the redox-active MLCT state. Similar concepts were applied in a series of ruthenium complexes studied by Han and co-workers, where they enhanced the cross section by increasing the number of highly conjugated ligands.^15^ These ruthenium(ii) complexes also promote a variety of photoredox reactions, including hydrodehalogenation, redox neutral cyanation reactions of tetrahydroisoquinoline, and metallaphotoredox reaction. TPA can also be attained through consecutive absorption of two photons. In 2019, Wenger's group introduced a novel approach to achieve TPA by involving triplet excited states as intermediates.^52^ While their work utilized high-energy blue light in contrast to the red or NIR light employed in previous studies, the use of a cheap continuous wave laser, rather than the expensive pulsed femtosecond/picosecond lasers required for simultaneous two-photon absorption, has made this approach more accessible for laboratory applications.

**Fig. 8:**
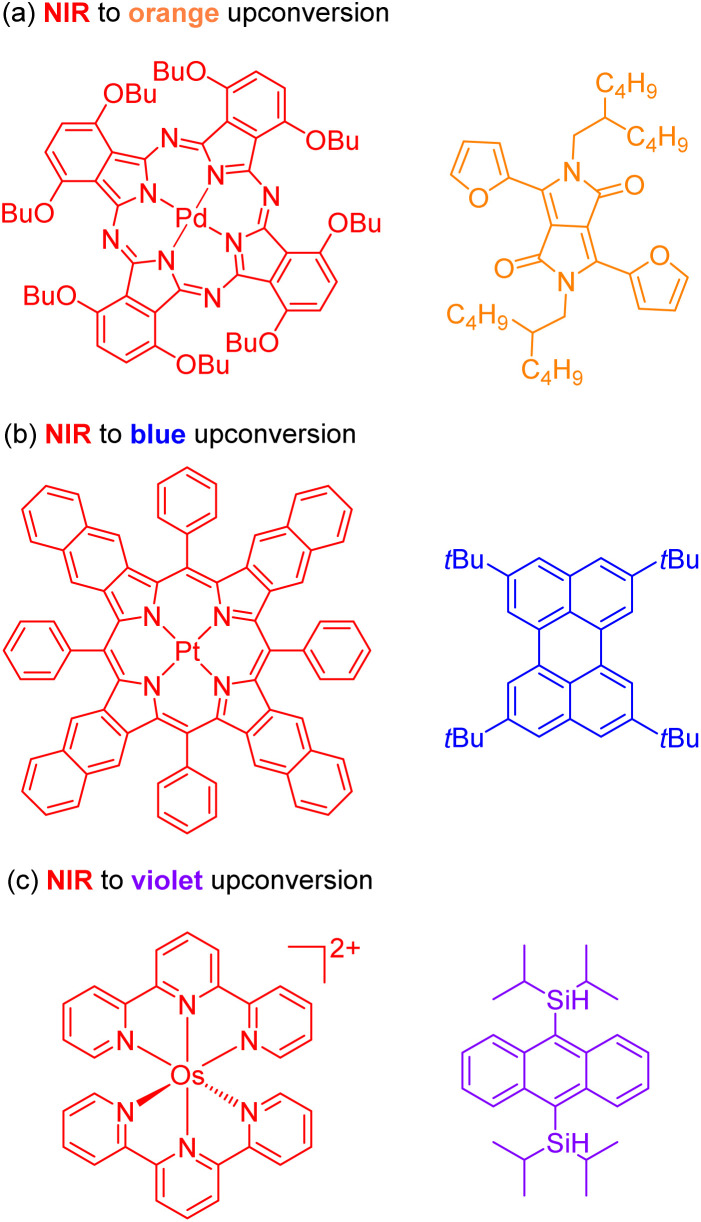
The control of output light *via* changing the sensitizers and annihilators. (a) NIR to orange upconversion. (b) NIR to blue upconversion. (c) NIR to violet upconversion.

The second approach to access the long-wavelength activation of a photoreaction is triplet–triplet annihilation upconversion (TTA-UC). The photophysical principle of the TTA-UC process is the formation of one high-energy photon (shorter wavelength) from the two low-energy photons (longer wavelength) *via* a bimolecular process involving a sensitizer (Sen) and annihilator (An) (Fig. 6b). To achieve efficient TTA-UC, the energy mismatch between the triplet state of the sensitizer and annihilator is a crucial requirement. In 2020, Huang and co-workers have shown that the triplet state energy of the annihilator perylene could decrease through increasing the π-conjugation, thereby influencing the reactivity of the catalytic system.^53^ To control the output light color, Ravetz and collaborators combined different sensitizers and annihilators to achieve orange and blue output light from red or NIR input light (Fig. 8a and b).^54^ They applied these upconversion schemes to a variety of catalytic reactions, where the excited annihilator (^1^[An]* in Fig. 6) either transfers energy to a conventional photocatalyst or directly participates in electron transfer with one of the substrates. To further increase the annihilator's output light energy, Haruki and co-workers have substituted anthracene with diisopropylsilyl groups at the 9,10-positions (Fig. 8c).^55^ As a result, the annihilator gave rise to violet fluorescence (415 nm) when combined with an appropriate sensitizer under NIR irradiation. Very recently, Glaser and Wenger combined the two strategies of TTA-UC and spin-forbidden transitions, using [Os(bpy)_3_](PF_6_)_2_ as a sensitizer and 9,10-dicyanoanthracene (DCA) as an annihilator to trigger a range of transformations.^56^ Besides precious transition metals such as Os, Pt, and Pd, there has been significant interest in exploring alternative complexes based on earth-abundant metals such as Mo, Zr, and Cr for TTA-UC applications.^57–59^

**Fig. 9:**
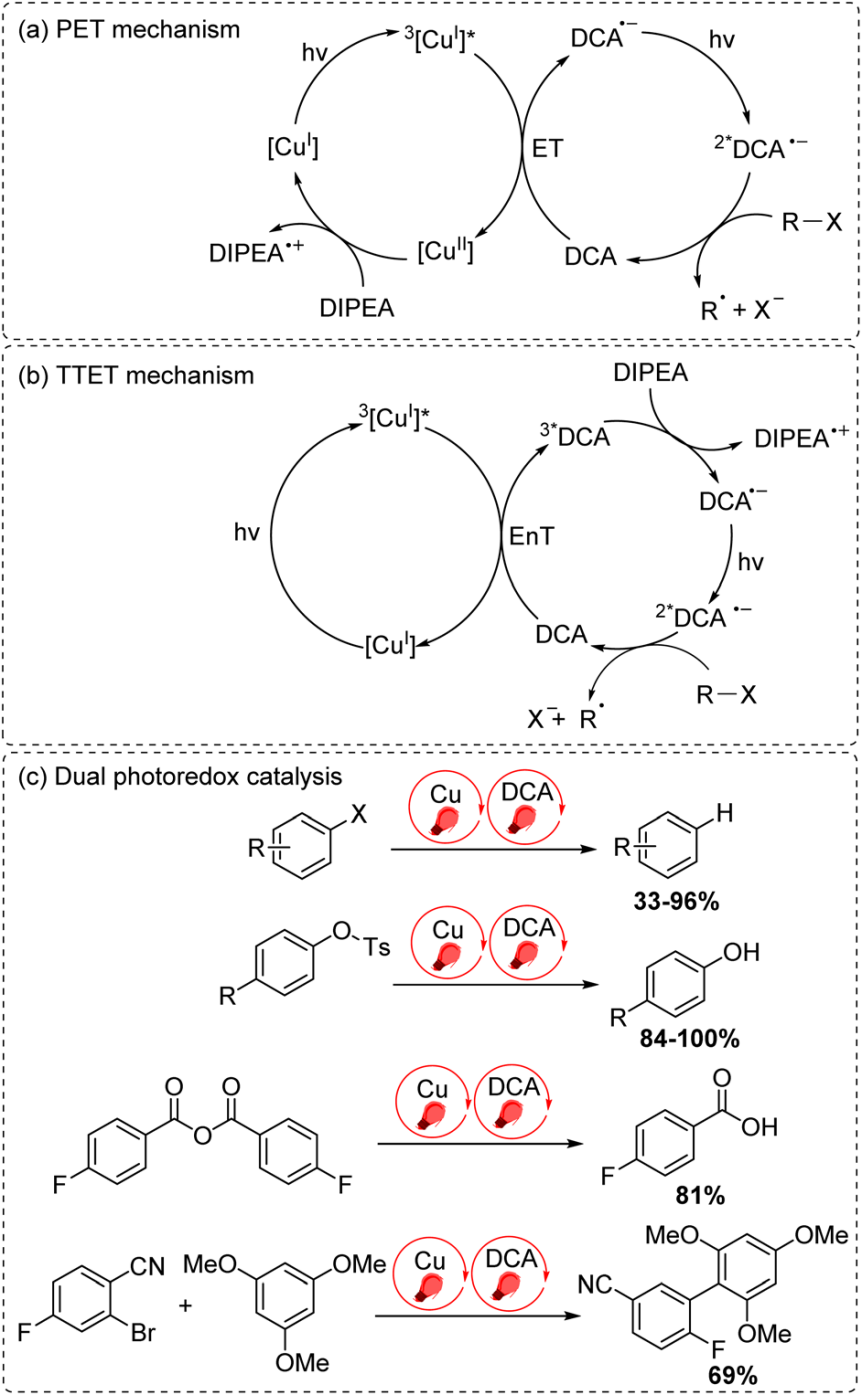
Dual photoredox catalysis driven by red light (632 nm). (a) PET mechanism. (b) TTET mechanism. (c) Dual photoredox catalysis.

The final approach to multiphoton catalysis described in this section is the emerging concept of dual photoredox catalysis, where two different photocatalysts are independently excited (Fig. 6c). This approach was recently reported by Glaser and co-workers, using [Cu(dap)_2_]Cl (dap = 2,9-dianisyl-1,10-phenanthroline) and DCA as photosensitizers with red light irradiation (*λ* = 632 nm).^16^ They proposed two mechanisms, a photoinduced electron transfer (PET) mechanism (Fig. 9a) and a triplet–triplet energy transfer (TTET) mechanism (Fig. 9b), for these transformations. Through this strategy, they have successfully photocatalyzed several reaction types such as dehalogenation, detosylation, cross-coupling reactions, and C–O bond cleavage reactions, which typically wouldn't operate under red light irradiation (Fig. 9c).

## Excited-state redox potentials

### Definitions and terms

To accomplish challenging redox transformations photochemically, it is necessary to have photosensitizers that are strong reductants or oxidants in their excited state. We point readers to a recent review article from our group that gives many specific examples of strategies to access strong excited-state reductants or oxidants, *i.e.* those we termed as having “extreme” redox potentials.^60^ Given the overlap with our recent review, this section will be intentionally brief, designed to provide a broad overview of the topic and emphasize our own contributions in this area.

To quantify how strong a photoreductant or photooxidant is, there are two relevant excited-state redox potentials to consider. The estimation of these potentials comes from modified Latimer diagrams^3^ that include the excited-state energy and the ground-state redox potentials. The excited-state energy (*E*_0,0_) can be determined in a variety of ways from photoluminescence measurements, and the ground-state potentials (*E*^red^ or *E*^ox^) come usually from cyclic voltammetry (CV) or differential pulse voltammetry (DPV) measurements. Eqn (1) and (2) can thus be used to estimate the excited-state potentials, where *E^ox^ is the potential for transferring an electron from the excited state and *E^red^ is the potential for accepting an electron. These equations should also each include an electrostatic work term (*w*) to be rigorously correct, which in some situations plays an important role,^61,62^ although the work term is often left out when estimating excited-state redox potentials for the purposes of photoredox applications. A more negative *E^ox^ defines a stronger photoreductant, and a more positive *E^red^ defines a stronger photooxidant.

1 *E^ox^ = *E*^ox^ – *E*_0,0_

2 *E^red^ = *E*^red^ + *E*_0,0_

The ground-state redox potentials of the one-electron oxidized or reduced form of the photosensitizer produced during the catalytic cycle should also be contemplated. These species can be involved in productive redox reactions with the substrate or co-catalyst, or if they react with a sacrificial reagent to close the photocycle their potentials dictate appropriate choices for the sacrificial reagent. For example, in the case of [Ru(bpy)_3_]^2+^, the redox couples are *E*_1/2_^III/II^ = +1.29 V *vs.* SCE (+0.89 V *vs.* ferrocene) and *E*_1/2_^II/I^ = −1.33 V *vs.* SCE (−1.73 V *vs.* ferrocene).^63^ Thus, the one-electron oxidized and reduced forms of [Ru(bpy)_3_]^2+^, formed respectively during an oxidative or reductive quenching event, are reasonably strong redox reagents in their own right.

### Methodological approaches

**Fig. 10:**
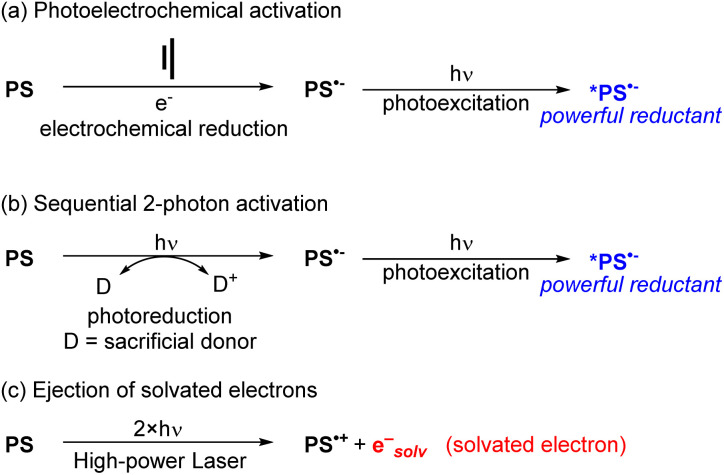
Summary of methodological approaches to access powerful visible-light photoreductants. (a) Photoelectrochemical activation. (b) Sequential 2-photon activation. (c) Ejection of solvated electrons.

To use visible light to generate a strong reductant or oxidant, there are two main strategies. The first is methodological in nature and involves using additional energy input to generate a potent excited-state redox reagent from a molecule that is not normally a particularly strong photooxidant or photoreductant. Representative examples of these approaches are summarized in Fig. 10, given in the context of reductive transformations, and again we refer the reader to our previous review for more detailed coverage and specific examples.^60^ Two of the approaches in Fig. 10 involve generation of photosensitizer radical anions (PS˙^−^), either electrochemically (Fig. 10a)^64,65^ or *via* sacrificial photoreduction (Fig. 10b).^66^ We do note that there is mechanistic controversy regarding the involvement of photosensitizer radical anions in productive photocatalytic pathways,^67,68^ but nonetheless combined electrochemical/photochemical activation and sequential two-photon activation have been successful at promoting photoredox transformations on substrates that are difficult to reduce. Finally, the third approach shown in Fig. 10c uses high-powered laser irradiation to enable consecutive two-photon activation of the photosensitizer, which in the case of certain MLCT photosensitizers can eject solvated electrons that serve as powerful reductants.^52^

### Photosensitizer design

The second major approach to accessing powerful visible-light photoreductants and photooxidants involves molecular design, controlling the two terms in eqn (1) and (2) to perturb the redox potentials to more extreme values. To keep this article centered on our own contributions we focus on powerful photoreductants, but there are likewise recent advances in photooxidant design.^60^ Either term in eqn (1) can be manipulated to increase excited-state reducing power, but it is less desirable to simply increase *E*_0,0_ since complexes with high excited-state energies typically are poor absorbers of visible light. Thus, it presents a significant challenge to design compounds that are excitable with visible light and are still strong photoreductants, and the advances presented in this section have succeeded in obtaining those two attributes together.

**Fig. 11:**
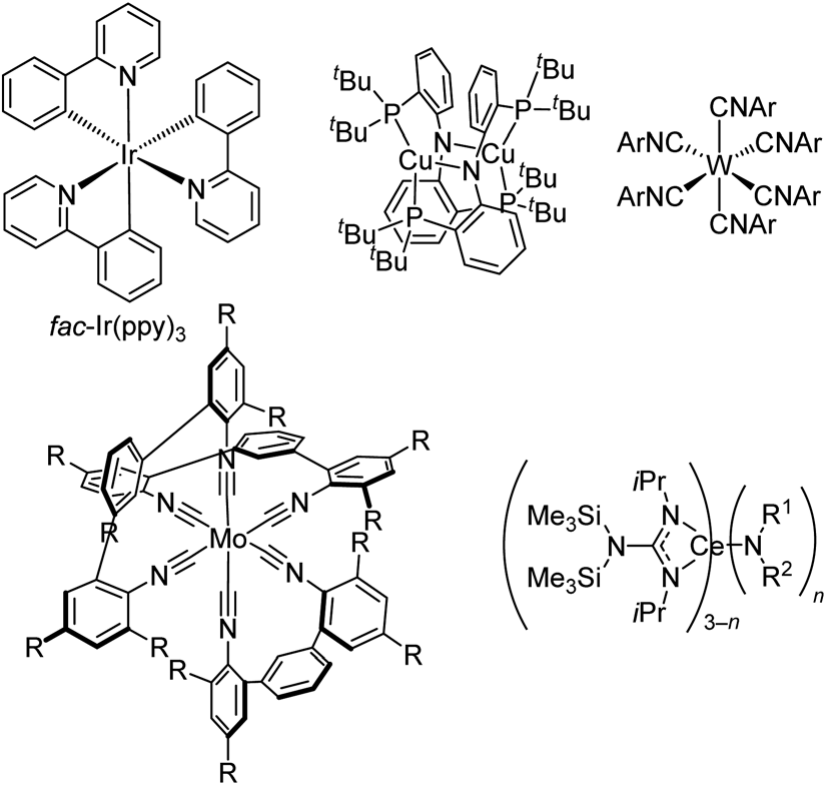
Representative examples of powerful visible-light photoreductants.

There are some organic photosensitizers that act as powerful photoreductants,^3,9,60^ but many of the fields of photoredox catalysis and solar fuels catalysis have been dominated by transition metal photosensitizers. Arguably one of the most widely used organometallic photosensitizers, particularly for challenging reductive transformations, is the homoleptic complex *fac*-Ir(ppy)_3_ (ppyH = 2-phenylpyridine, Fig. 11). It has an excited-state potential *E^ox^ of −2.1 V *vs.* ferrocene (−1.7 V *vs.* SCE), making it one of the strongest among the common organometallic photoreductants.

There have been two main approaches to accessing metal-based photoreductants that rival or exceed the reducing power of *fac*-Ir(ppy)_3_ and can offer complementary or superior reactivity in catalytic reactions. First, there are some groups that have investigated altogether new paradigms in metal complex design, moving beyond the cyclometalated iridium and ruthenium polypyridyl designs that have dominated. Fig. 11 also includes three classes of such compounds, albeit not intended to be a comprehensive listing of recently developed photoreductants. First introduced in 2005,^69^ Peters and co-workers have extensively developed dicopper phosphinoamide complexes like and related derivatives. This complex has *E^ox^ = −3.1 V *vs.* ferrocene, making it one of the strongest known photoreductants, and it has recently been shown to be able to promote radical homocoupling of benzyl chloride derivatives under 440 nm irradiation with electrochemical cycling.^70^ Another emerging class of photoreductants shown in Fig. 11 are homoleptic group 6 (Cr, Mo, and W) isocyanide complexes, either with six monodentate or three bidentate isocyanides. The W(CNAr)_6_ complexes, popularized by Harry Gray's group, have been shown to be exceptionally strong photoreductants,^71^ and their excited-state lifetimes can be extensively lengthened using oligoaryl and sterically encumbered isocyanides, allowing further optimization of their photosensitizer attributes.^72^ Some of these derivatives have been applied in photoredox intramolecular cyclization reactions of aryl iodide precursors,^51,73^ and as described in Fig. 7 some variants can be efficiently activated *via* two-photon absorption in near-infrared.^51^ Bidentate isocyanide analogues, primarily of Mo and Cr,^74–78^ have been extensively developed by Wenger's group, with some emerging applications in photoredox catalysis.^76–78^ As promising as these group 6 isocyanide complexes are, their applications as photocatalysts remain limited and have only been reported by the groups who originally synthesized the complexes. This likely originates from their challenging synthesis, which involves complex ligand synthesis (particularly in the case of tris–chelate complexes) and a demanding alkali metal reduction step to access the formally M(0) complexes, inhibiting their widespread adoption by practitioners of photoredox catalysis. To circumvent complicated syntheses of chelating isocyanide ligands and their M(0) complexes, Heinze's group reported a photoredox active molybdenum(0) carbonyl complex supported by a tripodal pyridine-based ligand that can be prepared in two steps.^79^ Finally, moving away from d-block elements altogether, Schelter's group has introduced cerium(III) complexes with mixed guanidinate/amide ligand sets and shown that some of these are strong photoreductants for photoredox catalysis. These compounds absorb deep blue light at 420 nm *via* a 4f → 5d electronic transition, and those with one amide and two guanidinate ligands (*n* = 1 in Fig. 11) are particularly effective in photoredox transformations on aryl halide substrates.^80,81^

**Fig. 12:**
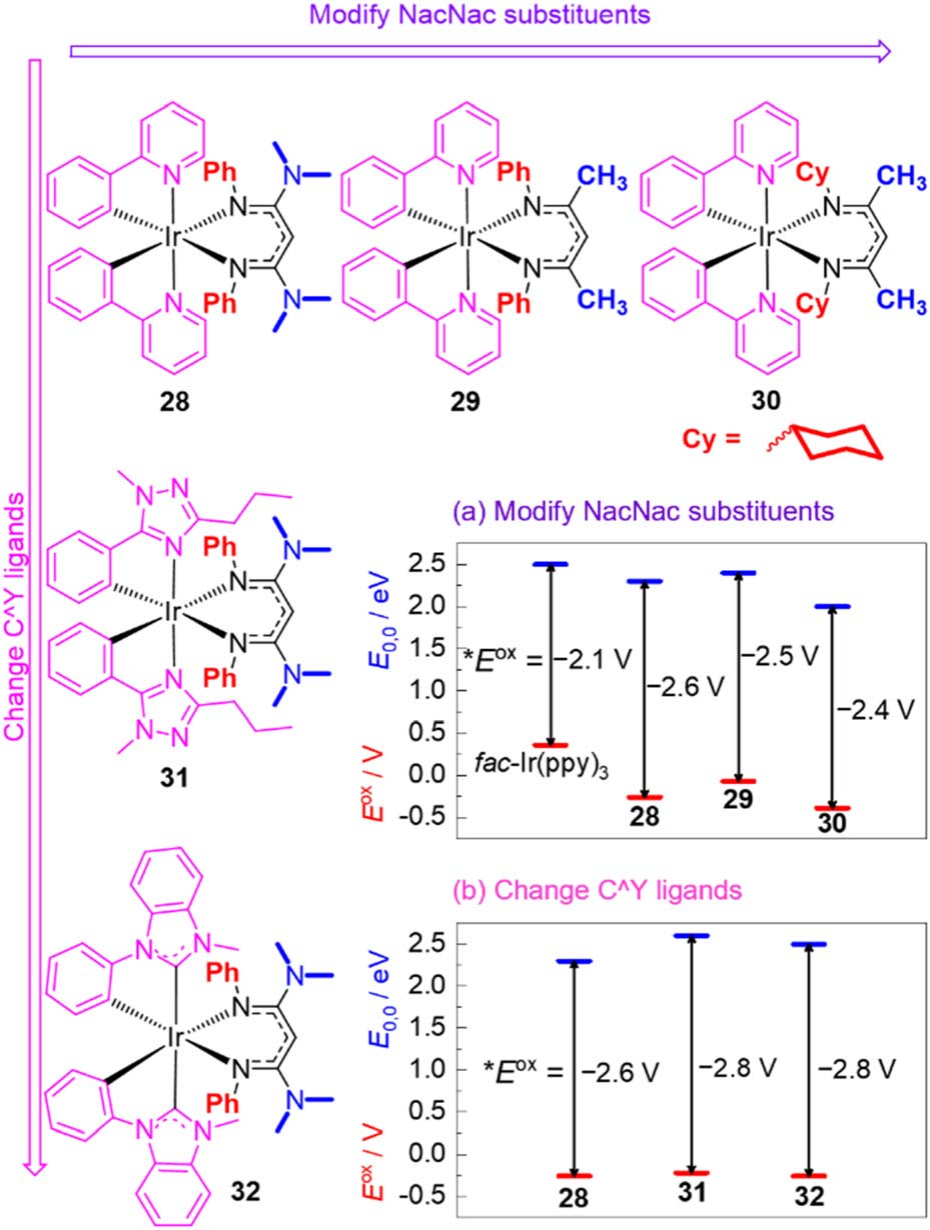
Summary of representative Ir(C^Y)_2_(NacNac) photosensitizers, demonstrating structure–property relationships involving *E*^ox^ (red lines) and *E*_0,0_ (blue lines). The *E*^ox^ values are referenced to the ferrocene couple, and the vertical arrows represent the absolute value of *E^ox^, as per eqn (1).

Recognizing their many attractive attributes, which include intense visible absorption, sufficiently long excited-state lifetimes, and good photostability, our group has taken the approach of modifying the well-known cyclometalated iridium structural motif to achieve more potent excited-state potentials. This approach is summarized in Fig. 12, which shows the structures of some of our recently developed iridium photosensitizers and graphically summarizes the effects on both terms in eqn (1), the ground-state redox potential *E*^ox^ and the excited-state energy *E*_0,0_. Modulation of these terms allows us to obtain *E^ox^ values substantially more negative than that of *fac*-Ir(ppy)_3_. Our strategy centers around replacing one of the ppy ligands in *fac*-Ir(ppy)_3_ with an electron-rich β-diketiminate (NacNac) ligand, furnishing heteroleptic compounds of the general form Ir(C^Y)_2_(NacNac), where C^Y is the cyclometalating ligand. These heteroleptic compounds offer two layers of control over the excited-state redox potential. Our initial efforts focused mainly on modifications to the NacNac ligand.^32,82^ As shown in the representative series 28–30 in Fig. 12, there are two positions on the NacNac ligand that can be easily varied, the *N*-substituents and the backbone groups. Fig. 12a shows how the two terms *E*^ox^ and *E*_0,0_ respond to the incorporation of NacNac ligands and their substitution pattern, with the difference between those two terms (vertical arrows) representing the absolute value of the excited-state potential *E^ox^, as per eqn (1). The data show that the NacNac ligand profoundly impacts the ground-state potential *E*^ox^, which shifts cathodically by at least 400 mV relative to *fac*-Ir(ppy)_3_. The excited-state energy *E*_0,0_ is much less perturbed, leading to 28–30 being more potent photoreductants than *fac*-Ir(ppy)_3_ by 300–500 mV. More significantly, these strongly photoreducing complexes allow for significantly faster rates of photoinduced electron transfer to electron-acceptor substrates like methyl viologen and benzophenone,^32^ and they promote a variety of photoredox transformations on challenging organic substrates, including unactivated aryl halides and ketones.^33,82,83^

Compounds 28–30 represent only a small subset of the Ir(ppy)_2_(NacNac) photosensitizers we have studied, but we found that because of a tradeoff between the two terms in eqn (1) (*E*^ox^ and *E*_0,0_), any modifications we made to the NacNac ligand or substituents we incorporated onto the ppy ligands yielded very similar *E^ox^ values in the range of −2.4 to −2.6 V. To obtain even more negative *E^ox^ values, we employed different classes of cyclometalating (C^Y) ligands, as shown in Fig. 12b. Both the triazole-based ligands in 31 and the NHC-based ligands in 32 cause a substantial increase in *E*_0,0_, with the ground-state potential still controlled by the NacNac ligand and varying little, which drives a *ca.* 200 mV shift in the excited-state potential.^84^ This increase in *E*_0,0_ does lead to a decrease in visible absorption relative to the C^Y = ppy analogues, but 31, 32, and related variants with the same C^Y ligands still have appreciable absorption beyond 400 nm and can be excited with blue light. The more negative excited-state redox potentials in 31 and 32 lead to faster rates of photoinduced electron transfer to benzophenone and acetophenone, although at present we have not realized any photocatalytic advantages with these more strongly photoreducing analogues.

## Improved excited-state lifetime

### Definitions and terms

**Fig. 13:**
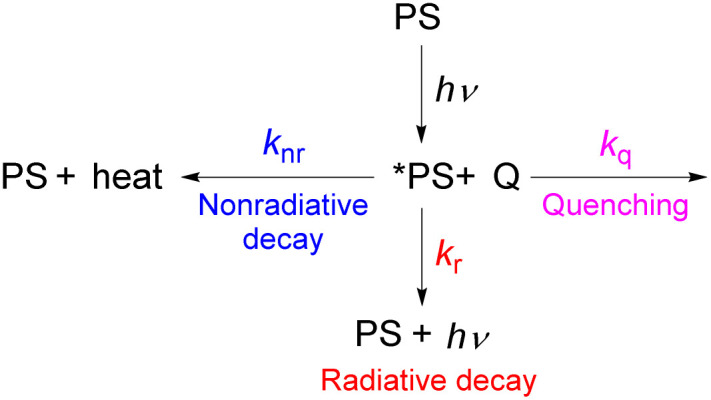
Possible excited-state decay pathways.

As described in the previous chapter, strong ground- or excited-state redox potentials are imperative to provide a positive driving force for electron transfer between photosensitizers and electron donors or acceptors. However, the kinetics and quantum yield (efficiency) of photoinduced electron transfer also depend on the photosensitizer's excited-state lifetime. After absorbing light, the photosensitizer (PS) enters an excited state (*PS), and there are a few parallel decay pathways, as illustrated in Fig. 13. Most photosensitization processes require bimolecular reactions, which means that the excited state needs to encounter the quencher molecule (*Q*) to transfer electrons or energy. If there is no bimolecular association between the photosensitizer and the quencher molecule in the ground state and the reaction between the two interacting chemical species (*PS and *Q*) is largely dominated by diffusion, as often occurs in the solution state, the process is termed a diffusion-limited or diffusion-controlled reaction. In general, the diffusion-limited rate constant in most organic solvents is about 10^9^ M^−1^ s^−1^.^85,86^ Since diffusion is a second-order process, the corresponding timescale is dependent on the concentration of the quencher that reacts with the photosensitizer's excited state, which is most often a substrate or sacrificial reagent. Given that many photoredox reactions are carried out at concentrations of 0.01–0.1 M in the substrate, diffusion would then occur on the timescale of tens to hundreds of nanoseconds. Therefore, the excited state should have a lifetime on the order of 10^−6^ to 10^−8^ s to ensure that bimolecular electron or energy transfer can compete with excited-state decay at reasonable quencher concentrations. In this section, we will highlight strategies for improving excited-state lifetimes and provide a few recent examples of transition metal-based photosensitizers with extended lifetimes.

### Steric strategies

**Fig. 14:**
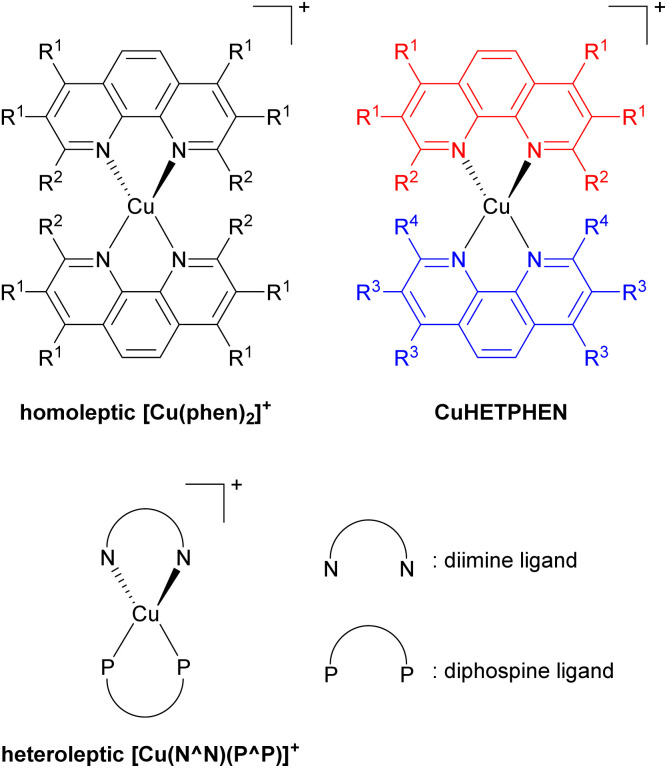
General structures of copper(i) complexes.

Low-cost replacements for expensive and rare transition metals such as iridium(iii), ruthenium(ii), and platinum(ii) are needed for sustainable applications, spurring the development of many cheap first-row transition metal-based photosensitizers. Of many potential candidates, copper(i) has been extensively developed, specifically the [Cu(phen)_2_]^+^ series, where phen is a 1,10-phenanthroline derivative as illustrated in Fig. 14. However, this bis-phenanthroline copper(i) motif undergoes structural distortion in the excited state, resulting in deactivation of the MLCT state and short excited-state lifetime.^87–89^ Pioneering studies from McMillin's group in 1980s and 1990s showed that introducing bulky substituents onto the 2- and 9-positions of the phenanthroline ligand can effectively restrict this structural distortion in the excited state and lead to longer excited-state lifetime.^90–95^ They also showed cooperative effects of additional methyl groups in 3- and 8-positions which boost the influence of the substituents in the 2- and 9-positions in the subsequent work.^96^ This increase in lifetime occurs because the alkyl substituents physically block the flattening Jahn–Teller distortion that occurs in the MLCT excited state, which is the origin of the short lifetimes in unsubstituted analogues. After that, Castellano's group furthered this insight by introducing a diverse range of alkyl groups onto phenanthroline, not only at the 2- and 9-positions but also at 3, 4, 7, and 8-positions.^97–100^

Copper(i) heteroleptic bis(1,10-phenanthroline) complexes, also known as CuHETPHEN, employ two different phenanthroline scaffolds and have been investigated by Mulfort and colleagues. Their general structure is also shown in Fig. 14.^101,102^ As the steric bulk of each phenanthroline moiety increases, the lifetime increases from 0.73 ns up to 74 ns. Heteroleptic [Cu(N^N)(P^P)]^+^ complexes, where N^N is a phenanthroline derivative or a diimine ligand and P^P is a diphosphine ligand, are another extensively studied class of copper(i) photosensitizers. In 2013, Mejía and colleagues reported heteroleptic copper(i) complexes using phenanthroline and diphosphine ligands.^103^ Within the series of complexes with the same diphosphine ligand, the excited state lifetime correlated with the steric bulk of the substituents on the phenanthroline ligand. Zhang reported similar results.^104^ They prepared heteroleptic [Cu(N^N)(PPh_3_)_2_]^+^ and [Cu(N^N)(P^P)]^+^ complexes and demonstrated the relationship between the steric bulk of each ligand and the photophysical properties. When methyl and butyl groups were introduced into the 2- and 9-positions of the phenanthroline ligand, the lifetime increased by approximately fourfold.

**Fig. 15:**
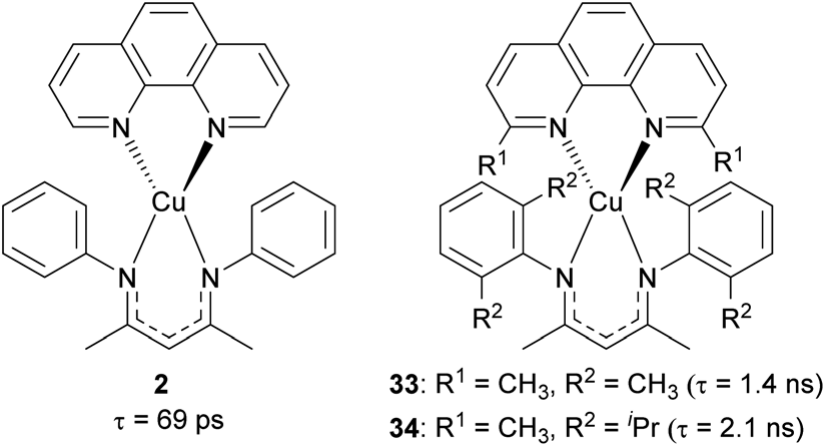
Structures of Cu(N^N)(NacNac^R^) complexes.

Our group recently reported heteroleptic copper(i) complexes with the general formula of Cu(N^N)(NacNac^R^), where N^N is the diimine ligand and NacNac^R^ is the β-diketiminate ligand, as summarized in Fig. 15.^105^ To increase the steric encumbrance, alkyl groups were introduced into each diimine and NacNac^R^ ligand. The parent complex 2 without alkyl substituents has a lifetime of only 69 ps, whereas complexes 33 and 34 bearing alkyl groups displayed 1.4 ns and 2.1 ns lifetimes, which are 20–30 times longer than that of 2. The increased lifetimes in complex 33 and 34 are attributed to sterically encumbered ligands, suggesting that the excited-state dynamics could be affected by the steric environment.

**Fig. 16:**
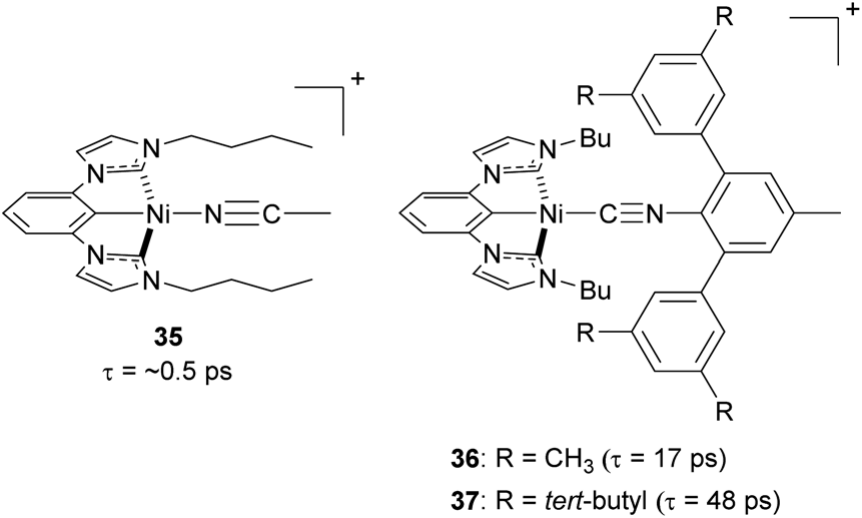
Structures of Ni(ii) complexes.

Steric strategies can be employed not only in copper(i) complexes but also in nickel(ii) complexes. In 2022, Ogawa and co-workers reported three new nickel(ii) complexes using a tridentate chelating ligand having a central cyclometalated phenyl unit with two *N*-heterocyclic carbenes, with the fourth site occupied by a monodentate isocyanide ligand (35–37, Fig. 16).^106^ The tridentate ligand provides the complex with rigidity and bulky substituents on the isocyanide ligand can play a role in preventing the structural distortion and protecting the metal center from nucleophilic attack in the axial direction. As a result of the improved steric protection afforded by the bulky isocyanide substituents, complexes 36 and 37 exhibit longer excited-state ^3^MLCT lifetimes (17 ps and 48 ps, respectively) than complex 35 (*ca.* 0.5 ps).

### Triplet reservoir

**Fig. 17:**
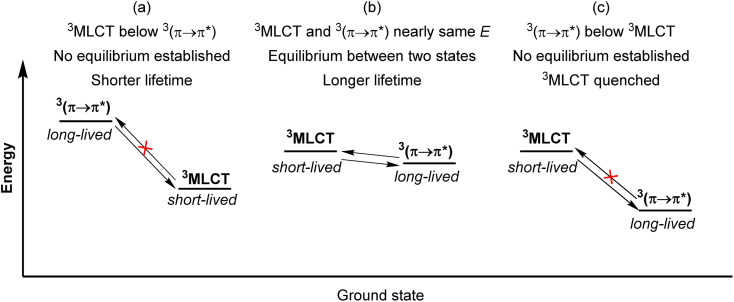
Simplified qualitative energy level diagrams illustrating the triplet reservoir effect. (a) Typical situation with ^3^MLCT below ^3^(π → π*). (b) Triplet equilibrium between ^3^MLCT and ^3^(π → π*). (c) Quenching of ^3^MLCT by lower-energy ^3^(π → π*).

**Fig. 18:**
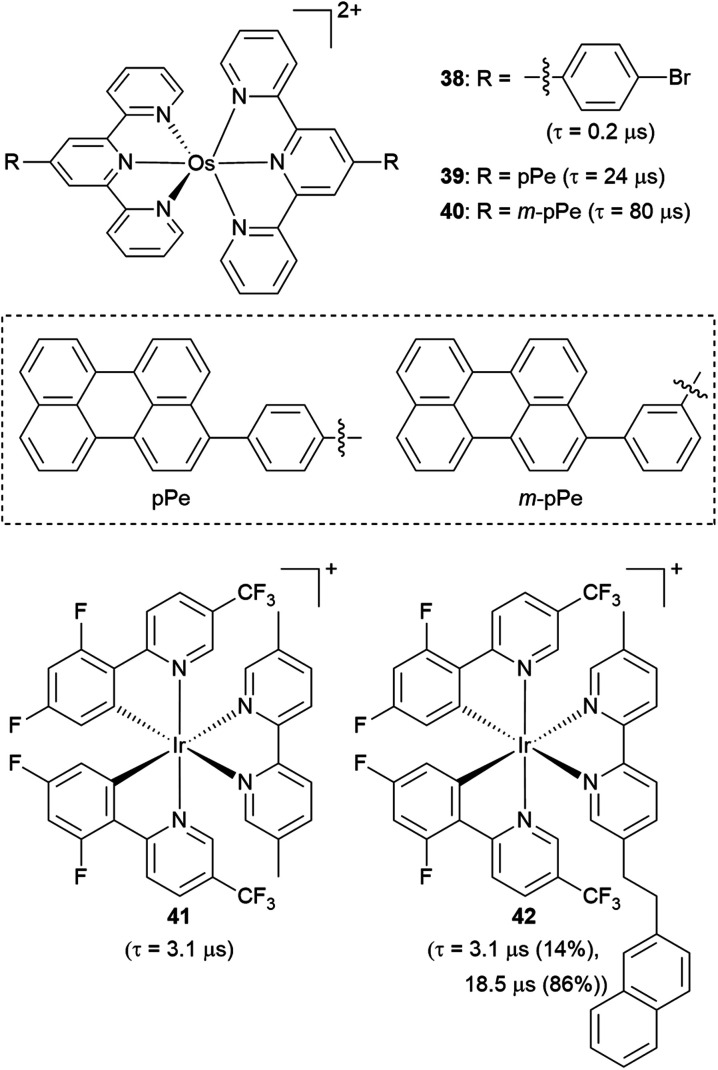
Osmium(ii) and iridium(iii) complexes with pendant organic chromophores.

Another strategy to increase the lifetime of metal-based photosensitizers is to covalently attach an organic chromophore to the periphery. Conventional conjugated ligands present ^3^(π → π*) states that are higher in energy than the ^3^MLCT state, in which case the system irreversibly populates ^3^MLCT upon excitation and all photochemistry arises from there (Fig. 17a). However, if the aromatic moiety's ^3^(π → π*) is energetically proximate to the ^3^MLCT state, an equilibrium between the two states can be established, as illustrated in Fig. 17b and first demonstrated by Ford and Rogers in 1992.^107^ The excited state “parks” in the long-lived, lower-lying ^3^(π → π*) state, which can repopulate the redox-active and emissive ^3^MLCT. This process is termed ‘triplet reservoir’ or, when luminescence occurs, thermally activated delayed photoluminescence (TADPL).^108^ If the ^3^MLCT energy level is higher than the ^3^(π → π*) state and the energy difference between the two state is larger than a few kcal mol^−1^ or 0.15 eV, the ^3^MLCT state could be quenched (Fig. 17c).^109,110^ The triplet reservoir effect in ruthenium(ii) complexes containing covalently attached aromatic chromophore units was extensively demonstrated by multiple groups in the 1990s and 2000s,^109–118^ and since then Castellano's group has investigated related triplet–triplet equilibria in rhenium(i),^119,120^ platinum(ii),^121^ and iridium(iii) complexes^122^ by combining them with various naphthalimide-substituted ligands. Along these same lines, Kimizuka's group has prepared osmium(ii) bis(terpyridine) complexes decorated with perylene subunits (38–40, Fig. 18).^123,124^ They observed the excited-state lifetime increasing to 24 μs in 39, compared to 0.2 μs in 38, resulting from a triplet-state thermal equilibrium between the phenyl-perylene (pPe) ^3^(π → π*) state and the osmium-centered ^3^MLCT state.^123^ In subsequent work, they observed further extension of the lifetime by changing the position of perylene from *para* to *meta* (40, *τ* = 80 μs).^124^

Li and coworkers recently prepared a naphthalene-decorated iridium(iii) complex (42, Fig. 18).^125^ The iridium(iii) complexes that they prepared (41 and 42) have higher triplet energies than most of the Ru(ii)-pyrene bichromophores. Due to the higher-lying triplet state, naphthalene was required to achieve the triplet reservoir effect. Complex 41 without the naphthalene unit showed a lifetime of 3.1 μs, whereas complex 42 exhibited a bi-exponential decay with 3.1 μs (14%) and 18.5 μs (86%), resulting from triplet–triplet equilibrium between the iridium(iii)-centered ^3^MLCT state and the ^3^(π → π*) state of the naphthalene unit. Recently, Bertrams and colleagues reported pyrene-decorated ruthenium(ii) complexes, taking advantage of the situation in Fig. 17c to switch between electron-transfer and energy-transfer pathways.^126^ The parent ruthenium(ii) complexes undergo electron transfer in the excited state, whereas in the pyrene-decorated complexes the low-lying pyrene ^3^(π → π*) state quenches ^3^MLCT and enables efficient energy transfer between the complex and quencher molecules.

### Molecular ruby spin-flip emitter

**Fig. 19:**
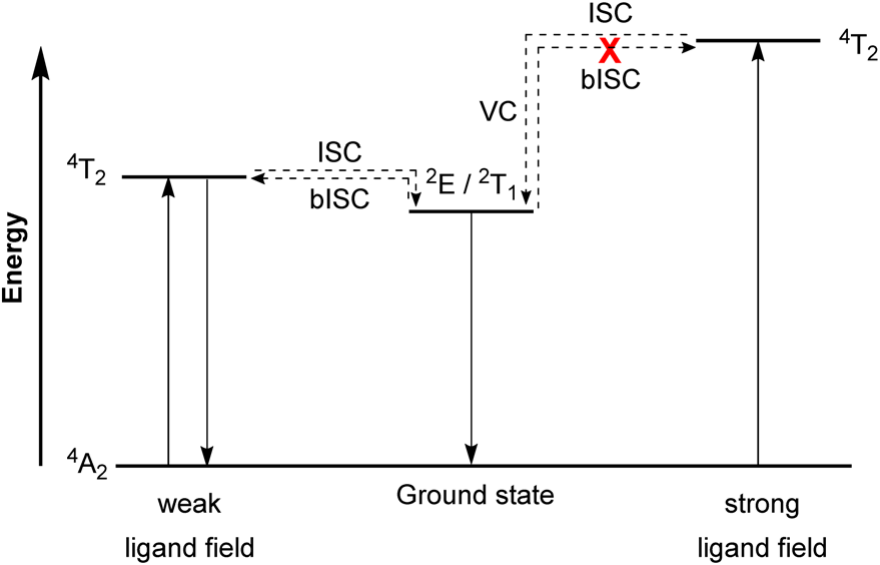
Simplified Jablonski diagram representing radiative processes of the spin-flip emitter. (ISC = Intersystem crossing, bISC = back-intersystem crossing, and VC = vibrational cooling).

In contrast to emissive charge-transfer (CT) transitions, metal-centered (MC) d–d states are typically nonradiative, which is especially problematic for 3d-metals with weak ligand fields, where these low-lying states facilitate nonradiative relaxation pathways to the ground state.^127,128^ Researchers have recently demonstrated intriguing phosphorescence in chromium(iii) complexes having emissive MC states. The luminescence in these compounds is referred to as “spin-flip (SF) emission” because emission occurs *via* a doublet to quartet transition that does not involve an electron transitioning between the e_g_ and t_2g_ orbitals. Compounds of this type have been nicknamed “molecular rubies”^129^ because of their analogy to the luminescent transition that is responsible for the characteristic red luster of naturally occurring mineral ruby. For trivalent chromium with a d^3^ electron configuration in an octahedral (O_h_) ligand field, the energy levels of the ^4^A_2_ ground state and the ^2^E, ^2^T_1_, ^2^T_2_, and ^4^T_2_ excited states should be considered. As illustrated in Fig. 19, after the excited state ^4^T_2_ is populated *via* spin-allowed excitation, transition to the ^2^E or ^2^T_1_ states occurs through intersystem crossing (ISC). In a weak ligand field, the energy levels of ^4^T_2_ and ^2^E or ^2^T_1_ states are close to each other and it possibly leads to back-intersystem crossing (bISC), resulting in fast relaxation to the ground state (^4^T_2_ → ^4^A_2_). To prevent bISC from occurring, a larger energy gap between the ^2^E/^2^T_1_ and ^4^T_2_ states is required. From the Tanabe–Sugano diagram of the d^3^ electron configuration,^130^ the energy of the ^4^T_2_ state is directly proportional to *Δ*_o_, whereas ^2^E and ^2^T_1_ states have a very shallow dependence on *Δ*_o_. Therefore, a strong ligand field is necessary to achieve a large *Δ*_o_ splitting and give a large energy difference between the ^4^T_2_ and ^2^E/^2^T_1_ states that avoids bISC. As such, the ligand designs for molecular rubies mostly focus on maximizing orbital overlap between the chromium d orbitals and ligand orbitals to achieve a strong ligand field.

**Fig. 20:**
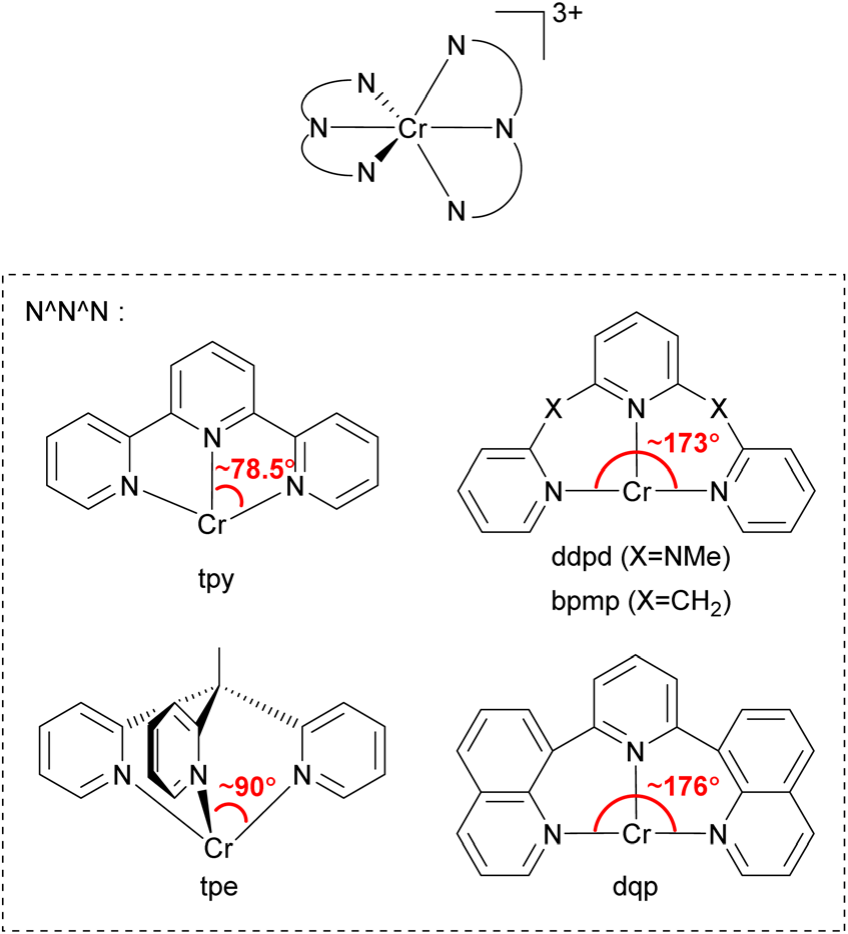
General structure of tridentate chelating ligands used to prepare homoleptic or heteroleptic chromium(iii) complexes.

In 2015, Heinze's group reported a highly near-IR-emissive homoleptic [Cr(ddpd)_2_]^3+^ complex, where ddpd is *N*,*N*′-dimethyl-*N*,*N*′-dipyridin-2-ylpyridine-2,6-diamine.^131^ Due to the *N*-Me spacer of the ddpd ligand, six-membered chelate rings with larger bite angles than terpyridine (tpy) are possible, as illustrated in Fig. 20, and it leads to nearly octahedral structures with strong metal–ligand orbital overlap, resulting in a 1164 μs lifetime in deaerated D_2_O. In order to achieve a nearly 90° bite angle, they also employed the tripodal 1,1,1-tris(pyrid-2-yl)ethane (tpe) ligand.^132^ The lifetime of *fac*-[Cr(tpe)_2_]^3+^ in H_2_O/HClO_4_ is 2800 μs, even longer than [Cr(ddpd)_2_]^3+^. Due to its long excited-state lifetime and photostability, [Cr(tpe)_2_]^3+^ exhibits photocatalytic activity in [4 + 2] cycloaddition of *trans*-anethole^132^ and fixation of SO_2_ into sulfones and sulfonamides.^133^

Piguet and coworkers employed the 2,6-di(quinoline-8-yl)pyridine (dqp) ligand to synthesize homoleptic or heteroleptic chromium(iii) complexes.^134–136^ As illustrated in Fig. 20, dqp has a *ca.* 176° N–Cr–N bite angle which is a slightly larger than that of the ddpd ligand (∼173°). They concluded that the larger bite angle can induce more effective metal–ligand orbital overlap and a larger ligand-field splitting.

Furthermore, the two fused aromatic quinoline units in the dqp ligand can reduce the degrees of freedom, limiting nonradiative relaxation pathways. [Cr(dqp)_2_]^3+^ has a lifetime of 1.2 ms at 293 K and 3 ms at 77 K in deaerated aqueous solutions.^134^ Bürgin and colleagues demonstrated the photocatalytic activity of [Cr(dqp)_2_]^3+^ in a couple of reactions including bromination of methoxyarenes, oxygenation of 1,1,2,2-tetraphenylethene, hydroxylation of arylboronic acids, and vinylation of an *N*-aryl amine.^137^

### Ligand design in Fe(ii) photosensitizers

**Fig. 21:**
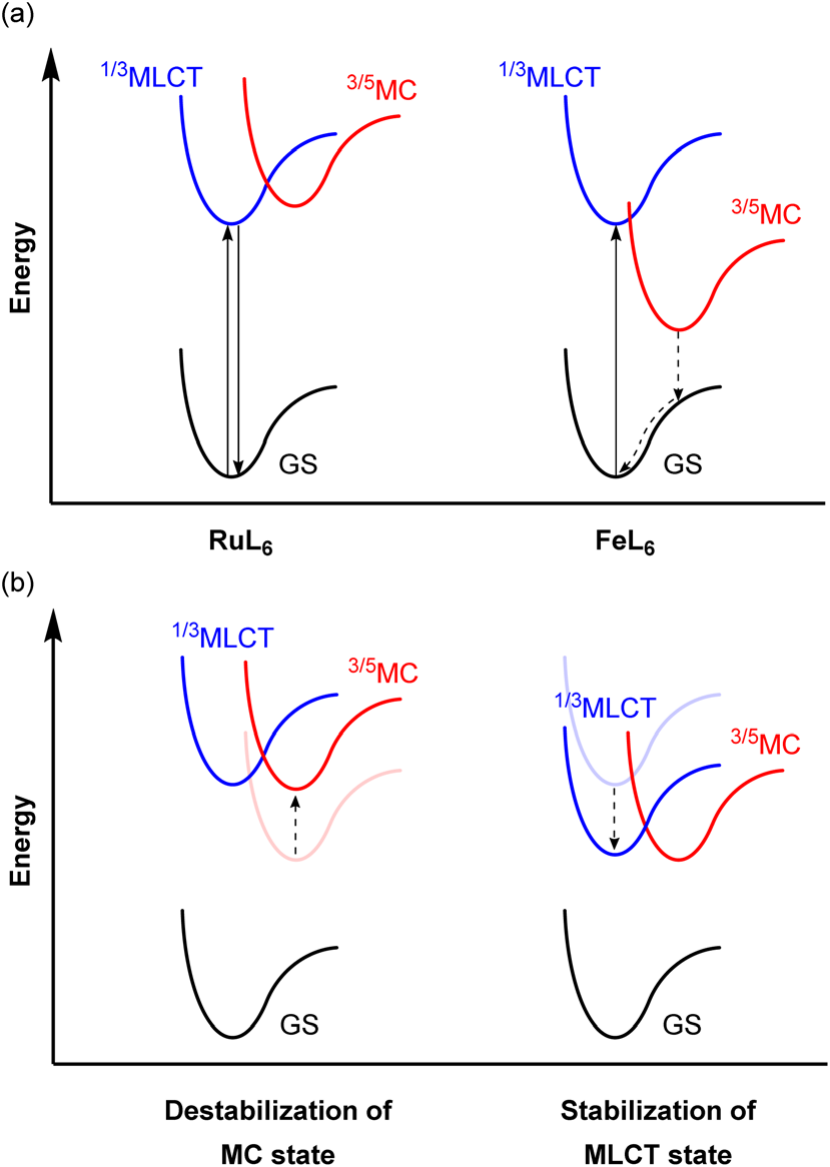
(a) Simplified potential energy surface diagrams of six-coordinate Ru(ii) and Fe(ii) complexes. (b) Strategies to achieve a larger energy barrier between MLCT and MC states.

Because of the ubiquity and wide-spread success of ruthenium(ii) polypyridyl complexes as photosensitizers, there is considerable interest in replacing ruthenium(ii) with isoelectronic iron(ii), due to the high abundance and low cost of iron. Even though they are isoelectronic (*d*^6^) and have the same six-coordinate octahedral geometry, the energy levels of the triplet metal-to-ligand charge transfer (^3^MLCT) and metal-centered (^3^MC) states are different from each other. The 4d orbitals of ruthenium(ii) more effectively overlap with the ligand orbitals, which induces a strong ligand field, whereas the smaller radial extension of iron(ii)'s 3d orbitals gives a much weaker ligand field. This difference in the ligand field strength provides different excited-state decay pathways as illustrated in Fig. 21a. In RuL_6_, MC states usually have higher energy than MLCT states, whereas these states are inverted in FeL_6_ complexes and provide detrimental non-radiative relaxation pathways, deactivating the MLCT state on the femtosecond timescale.^138–141^ For example, the activation energy for internal conversion from the MLCT to the MC state in the [Fe(bpy)_3_]^2+^ complex is only *ca.* 300 cm^−1^.^142^ To circumvent this fast relaxation pathway, the energy barrier between the MLCT and MC states should be larger. Although numerous strategies have been demonstrated and extensively reviewed,^127,143–146^ they can be roughly categorized into two approaches, illustrated in Fig. 21b: (i) destabilization of the MC state and (ii) stabilization of the MLCT state. Since Wärnmark and co-workers firstly demonstrated that *N*-heterocyclic carbene (NHC) ligands can significantly destabilize the ^3^MC state due to their strong σ-donating nature, giving iron(ii) chromophores with 9 ps MLCT lifetimes,^147^ many following studies have used NHC ligands. They also reported the complex [Fe(btz)_3_](PF_6_)_3_, where btz is 3,3′-dimethyl-1,1′-bis(*p*-tolyl)-4-4′-bis(1,2,3-triazol-5-ylidene).^148^ The low-spin d^5^ iron(iii) results in spin-allowed emission from a doublet ligand-to-metal charge transfer state (^2^LMCT), which is rarely observed in transition metal complexes, leading to an extended excited-state lifetime of 100 ps. Here we will provide other examples of iron photosensitizers with enhanced excited-state lifetimes.

**Fig. 22:**
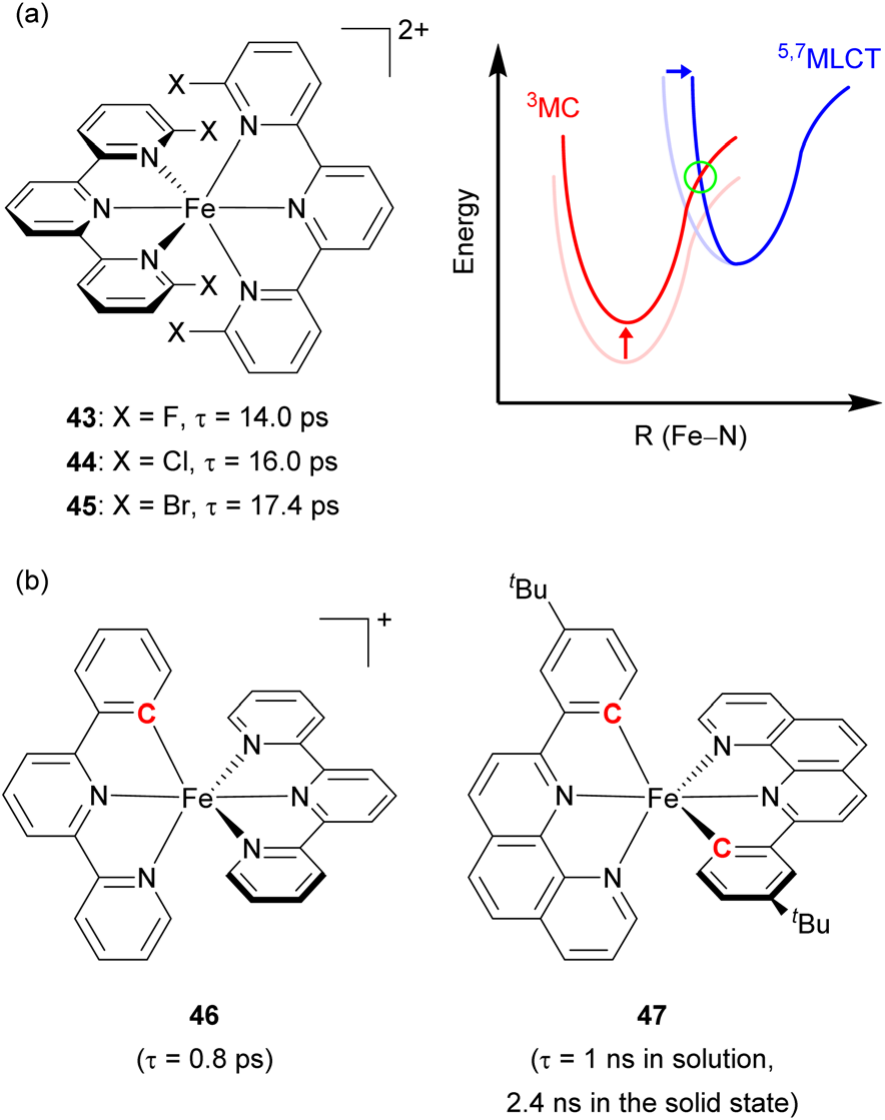
(a) Structures of Fe(ii) complexes with halogen-substituted terpyridine and the energy levels of MLCT and MC states. (b) Structures of cyclometalated Fe(ii) complexes.

Damrauer's group decorated the 2,2′:6′,2′′-terpyridyl ligand with halogen atoms (F, Cl, and Br, Fig. 22a) to fabricate a sterically demanding coordination environment.^149,150^ As illustrated in Fig. 22a, the increased energy barrier (in the green circle) was derived from both the destabilization of the MC state and the reorganization energy, inducing a longer MLCT lifetime across the halogenated series from 43 with 14 ps lifetime to 44 with 16 ps, and finally 45 with 17.4 ps. Steube and co-workers achieved a stabilized ^3^MLCT state by replacing one *N*-donor part of the terpyridine (tpy) ligand with a cyclometalating phenyl group (pbpy, 6-phenyl-2,2′-bipyridine) as shown in Fig. 22b (complex 46).^151^ This modification stabilized the ^3^MLCT state by 0.95 eV compared to the reference complex, [Fe(tpy)_2_]^2+^. In the reference complex the ^5^MC state was the finally populated excited state that causes a short lifetime of 145 fs, whereas 46 had a ^1^MLCT → ^3^MLCT → ^3^MC → ^1^A_1_ (GS) deactivation pathway due to its higher energy barrier, resulting in an increased lifetime of 0.8 ps.

The Berkefeld group added one more cyclometalating group to the iron(ii) complex (47, Fig. 22b), demonstrating double cyclometallation of a phenylphenanthroline framework.^152^ This double cyclometallation strategy significantly stabilized the ^3^MLCT state making ^3^MLCT the lowest excited-state. 47 exhibited a 1 ns lifetime in the liquid phase, showing photocatalytic activity in the stoichiometric radical C–halide/C–H cross coupling reaction. These above-mentioned two cases were successfully verified by computational studies,^153,154^ investigating the possibility that cyclometallation can stabilize the ^3^MLCT state and inhibit intersystem crossing between ^3^MLCT and ^5^MC, leading to an increase in excited-state lifetime.

## Conclusions and outlooks

Since molecular photosensitizers absorb light and convert it to a chemical potential, photosensitizers with superior photophysical properties are essential to use and convert solar energy efficiently. Although optimization of photocatalytic processes with already existing photosensitizers is still necessary, continuous development of new photosensitizers with improved properties is also imperative to expand photosensitizer libraries and access new photocatalytic reactivity. Of many photophysical properties, light absorption, redox potentials both in the ground and excited states, and the excited-state lifetime are key factors that are directly related to the photosensitizer's efficacy, as we discussed in this perspective. To capture a large portion of the solar spectrum, which is relevant for applications related to solar fuels, photosensitizers should absorb a wide range of the visible and even the near-IR region with a large molar absorption coefficient. Potent redox potentials, especially in the excited state, provide the thermodynamic driving force that can activate challenging substrates. From a kinetic perspective, sufficiently long excited-state lifetime enables photosensitizers to react with the substrate or quencher, facilitating bimolecular energy or charge transfer. Thus, the “ideal” photosensitizer has intense long-wavelength absorption, highly positive *E^red^ and/or highly negative *E^ox^ potentials, and a long lifetime, capable of driving challenging chemical reactions in synthesis and energy-related applications.

Unfortunately, optimization of all three parameters at the same time is nearly impossible since there are inherent trade-offs between each factor. For instance, a red-shifted absorption profile requires a smaller HOMO–LUMO gap, which normally gives a smaller *E*_0,0_ value and attenuates excited-state redox potentials as a result. In other words, increasing light absorption can reduce the charge-transfer driving force. Also, the low-energy excited states of photosensitizers that absorb longer wavelengths in the visible or near-IR region lead to short lifetimes according to the energy gap law.^155^ Our studies on heteroleptic copper(i) complexes, discussed in the ‘Improved light absorption’ section, serve as an example of this trade-off. These compounds absorb over a large portion of visible and near-IR regions, but they are not luminescent and have short excited-state lifetimes. In our subsequent study, we did show some improvement on this front, with bulky alkyl substituents on the diimine and NacNac ligands preserving the broad absorption out to ∼900 nm, while improving the excited-state lifetime from the picosecond to nanosecond timescale. In order to circumvent these inherent limitations, disruptive approaches are needed. As shown in this perspective, in some cases, fine-tuning of each parameter by modifying well-known structure types is an effective strategy for improving one or more of these factors. In other cases, methodological approaches lead to distinct improvements, where combinations of two inherently imperfect photosensitizers or a change in the reaction mechanism obviates these limitations. And finally, there are recent examples in this article of entirely new photosensitizer designs that go against conventional frameworks and severely disrupt these scaling relationships, *e.g.*, the molecular rubies discussed in the previous section that not only have very low-energy excited states but also have exceptionally long lifetimes.

Other factors that we didn't mention in this perspective might need to also be contemplated to overcome possible challenges. (i) *Chemical stability.* Photosensitizers should not react with any substrate in the ground state before being irradiated and should not rapidly decompose under the reaction conditions. (ii) *Photostability.* If the photosensitizer is unstable under irradiation, it likewise will underperform in catalytic applications. (iii) *Quantum yield.* Often overlooked when evaluating photocatalytic performance, the quantum yield is an important consideration when quantifying the efficiency of a photocatalytic reaction.^156^ The quantum yield depends on many reaction parameters, but the choice of the photosensitizer can be important to optimize the quantum yield. If the quantum yield for a photoreaction is low, an intense light source and/or a longer irradiation time would be needed to achieve suitable conversion.

While there is value in using “off-the-shelf” photosensitizers combined with methodology development and reaction engineering to drive advances in photocatalysis, we hope that this perspective convinces the reader that there are also many recent developments in photosensitizer design that have paved the way. Furthermore, it is necessary to consider and optimize multiple parameters when choosing a photosensitizer for a particular application or designing a new one for fundamental study or application. Since these parameters are intertwined *via* limiting scaling relationships it is normally difficult to optimize them all at once, but further advances in photosensitizer design should allow these limitations to be overcome or circumvented. As such, new creative approaches and continued fundamental investigations of photosensitizer candidates will play an important role in advancing the fields of photochemistry and photocatalysis.

## Author contributions

Dooyoung Kim: conceptualization, writing – original draft, writing – review & editing, and visualization. Vinh Q. Dang: conceptualization, writing – original draft, writing – review & editing, and visualization. Thomas S. Teets: conceptualization, writing – original draft, writing – review & editing, visualization, supervision, project administration, and funding acquisition.

## Conflicts of interest

There are no conflicts to declare.

## Supplementary Material
